# Data on horizontally transferred genes in California two-spot octopus, *Octopus bimaculoides*

**DOI:** 10.1016/j.dib.2018.05.132

**Published:** 2018-06-01

**Authors:** Liu Conghui, Liu Bo, Zhang Yan, Jiang Fan, Ren Yuwei, Li Shuqu, Wang Hengchao, Fan Wei

**Affiliations:** Agricultural Genomics Institute at ShenZhen, Chinese Academy of Agricultural Sciences, Shenzhen 518124, China

## Abstract

Horizontal gene transfer (HGT), a mechanism that shares genetic material between the host and donor from separated offspring branches, has been described as a means of producing novel and beneficial phenotypes for the host organisms. In the present study, 12 HGT genes were identified from California two-spot octopus *Octopus bimaculoides* based on a similarity search, phylogenetic construction, gene composition analysis and PCR (Polymerase Chain Reaction) validation. The data collected from the HGT genes from octopus, indicating the phylogenetic incongruences, CodonW analysis, PCR products, detailed motifs and organisms used in screening. In phylogenetic screening, those genes were nested within bacteria homologs and identified as HGT genes transferred from the bacteria to the octopus. The motifs were similar in proteins of the horizontally acquired Zn-metalloproteinases, but differed to endogenous proteins. CodonW was employed to investigate the codon usage bias between HGT genes and other genes in the octopus genome. In PCR validation, all the HGT genes could be produced as amplified fragments. The results collectively indicated the existence of HGT in molluscs and its potential l contribution to the evolution of octopus with regards to functional innovation and adaptability.

**Specifications Table**

**Table:** 

Subject area	*Biology*
More specific subject area	*Bioinformatics, Evolutionary biology*
Type of data	*Table, text file, and figure*
How data was acquired	*The phylogenetic trees were constructed by MEGA. The motifs were analyzed from MEME. The CodonW result was produced by CodonW. The sequences of HGT genes were sequenced by Sanger method.*
Data format	*The organism list, PCR primers and sequences were Raw. The phylogenetic trees, motifs and CodonW result were analyzed.*
Experimental factors	*33,638 protein-coding genes from Octopus bimaculoides, Protein sequences of 2774 bacteria, 26 protozoa, 50 fungi, 12 plants and 7 vertebrates were included for analysis*
Experimental features	*The HGT determination process was composed by three rounds of BLAST alignment and two rounds of phylogenetic analysis.*
Data source location	*All genomic sequences were collected from the NCBI and KEGG ftp site. Octopus bimaculoides for gene clone was collected from Shenzhen, Guangdong province, China.*
Data accessibility	*All the data are contained in this data article.*
Related research article	*Ancient Horizontally Transferred Genes in the Genome of California Two-Spot Octopus, Octopus bimaculoides (in press)*

**Value of the data**

• Molluscs are highly diverse and second only to arthropods in numbers, while the HGT studies are still insufficient. We report of the existence of HGT between bacteria and mollusc.
• 12 HGT genes were sifted out in the genome of octopus by the standard of phylogenetic incongruences, which were nested within bacteria homologs.
• PCR assay was performed to clone the cDNA fragments of HGT genes, validating the existence and expression of the HGT genes.
• The motifs were similar in proteins of the horizontally acquired Zn-metalloproteinases, but differed to endogenous proteins.

## 1 Data

**Fig. 1:**
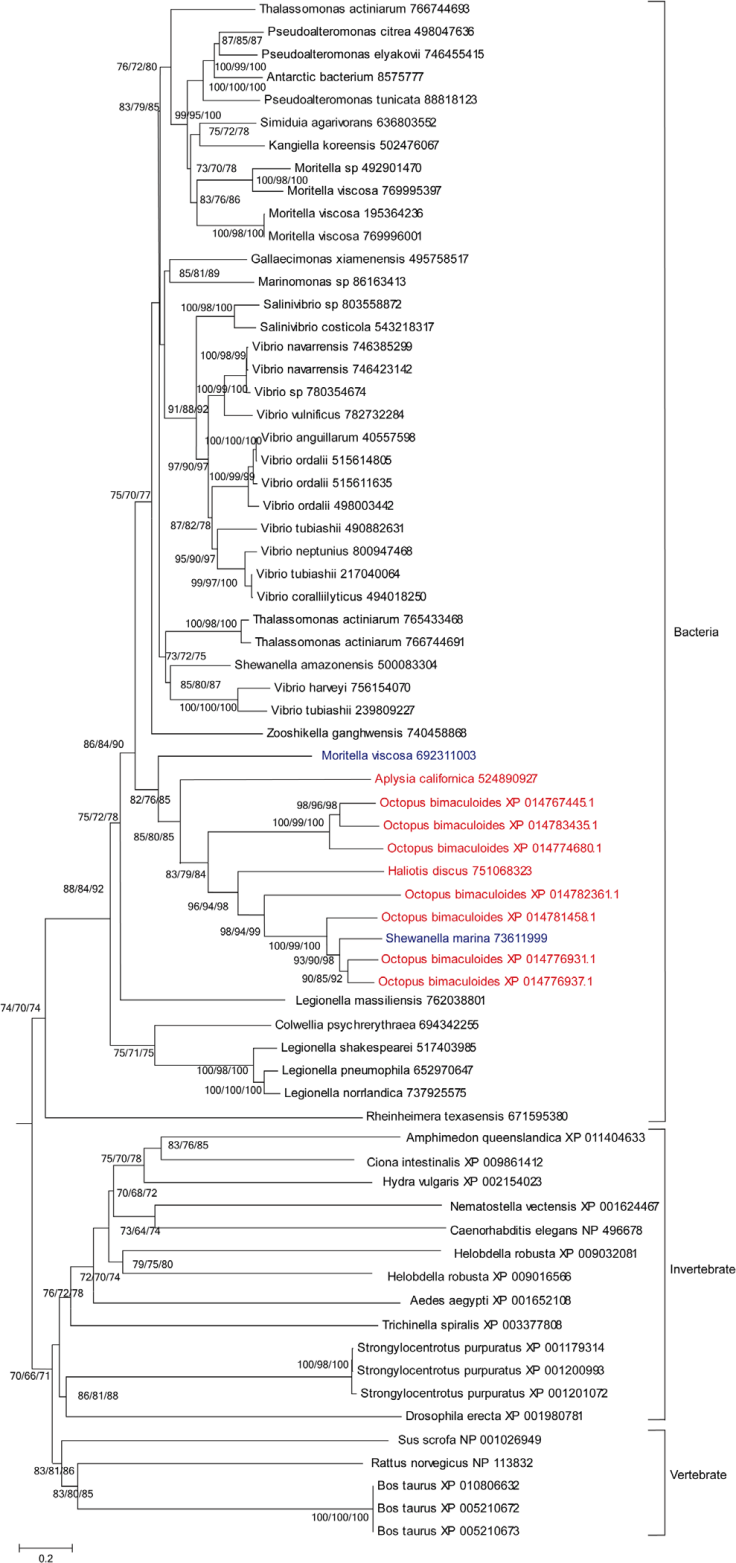
Phylogenetic analysis of the octopus ZnMPs and their homologues.

**Fig. 2:**
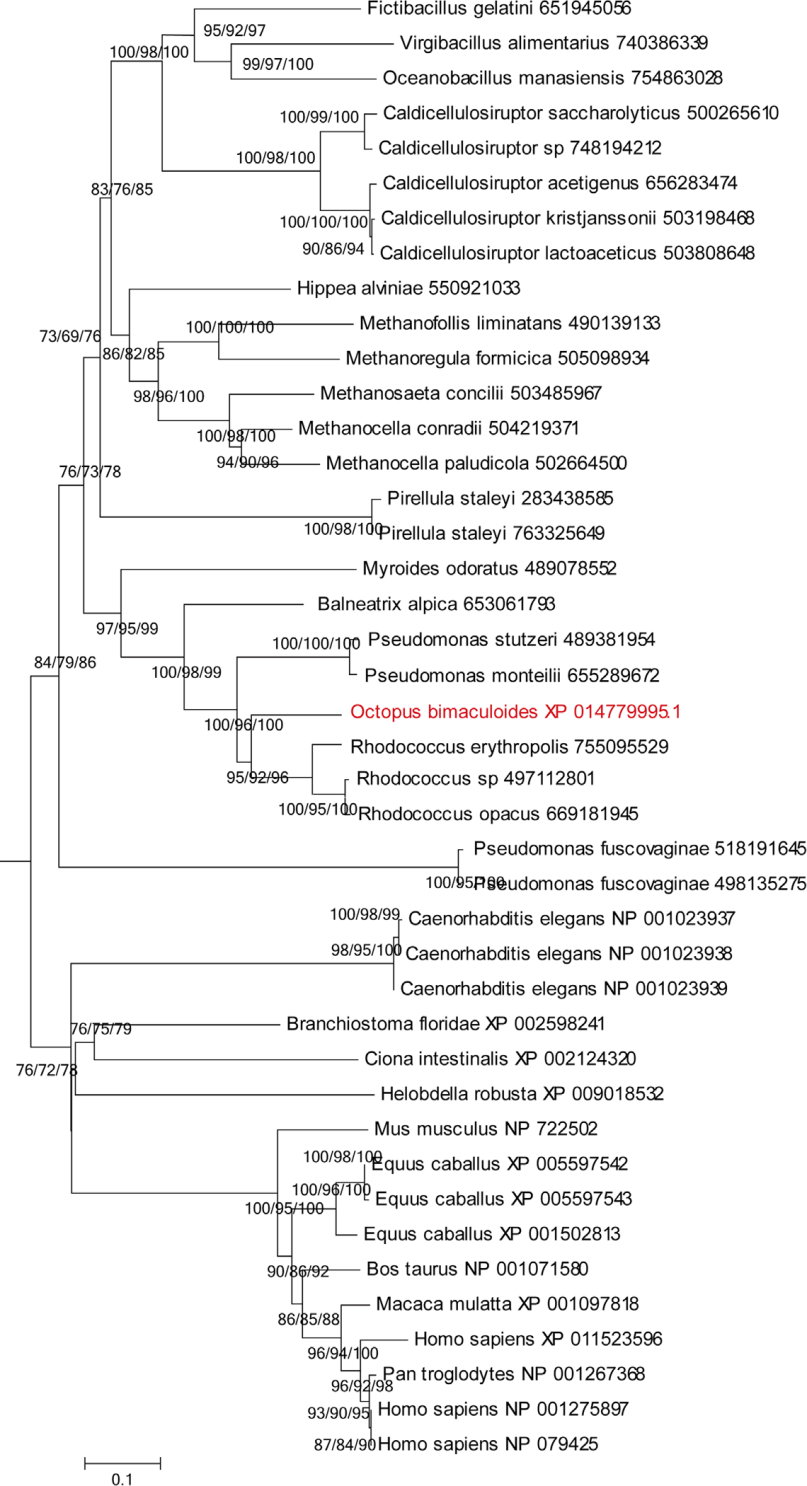
Phylogenetic analysis of the octopus D-AL (XP_014779995.1) and their homologues.

**Fig. 3:**
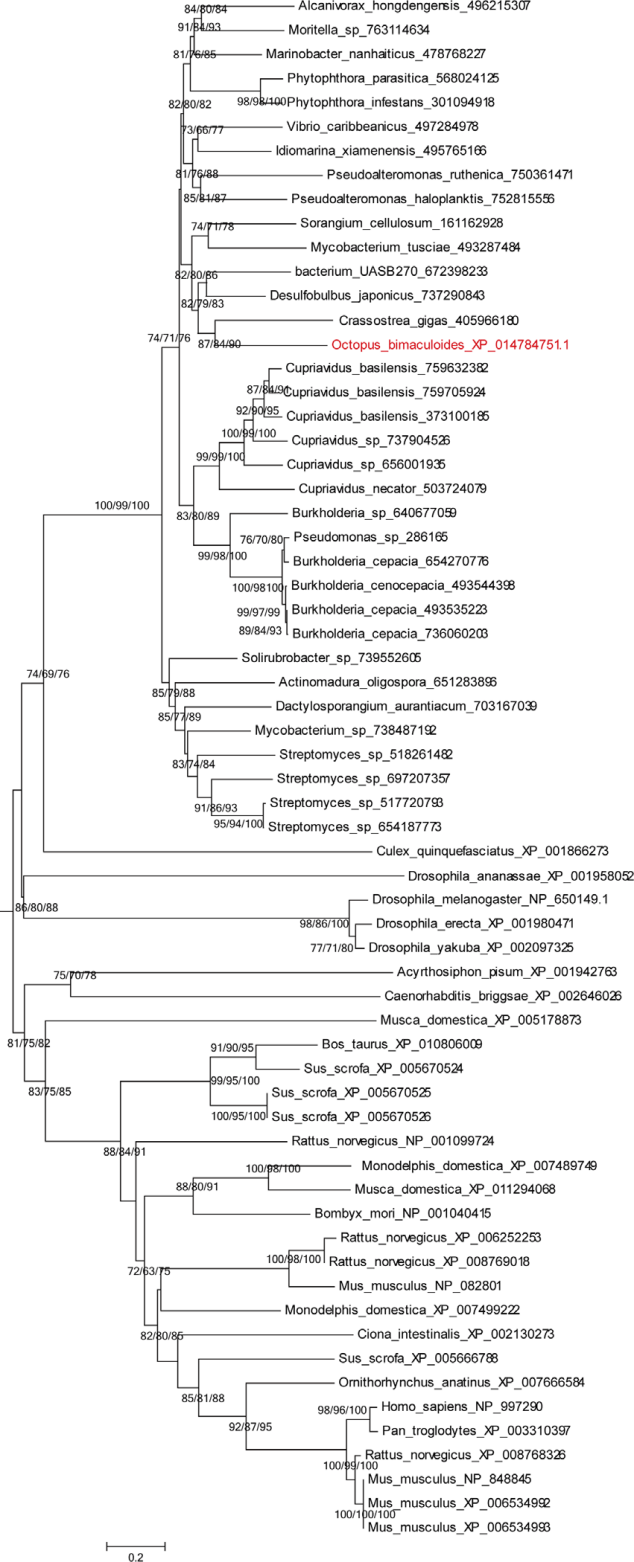
Phylogenetic analysis of the octopus PGS (XP_014784751.1) and their homologues.

**Fig. 4:**
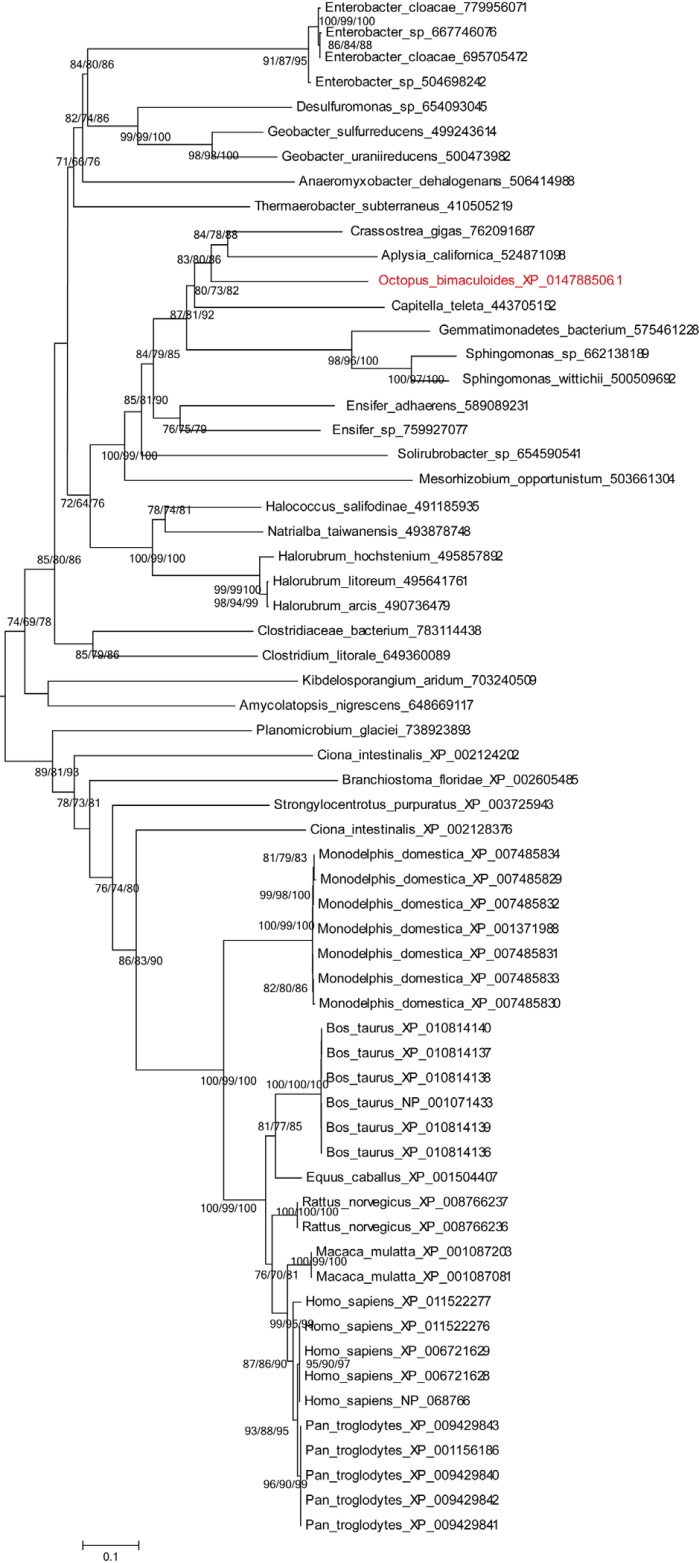
Phylogenetic analysis of the octopus SRL (XP_014788506.1) and their homologues.

**Fig. 5:**
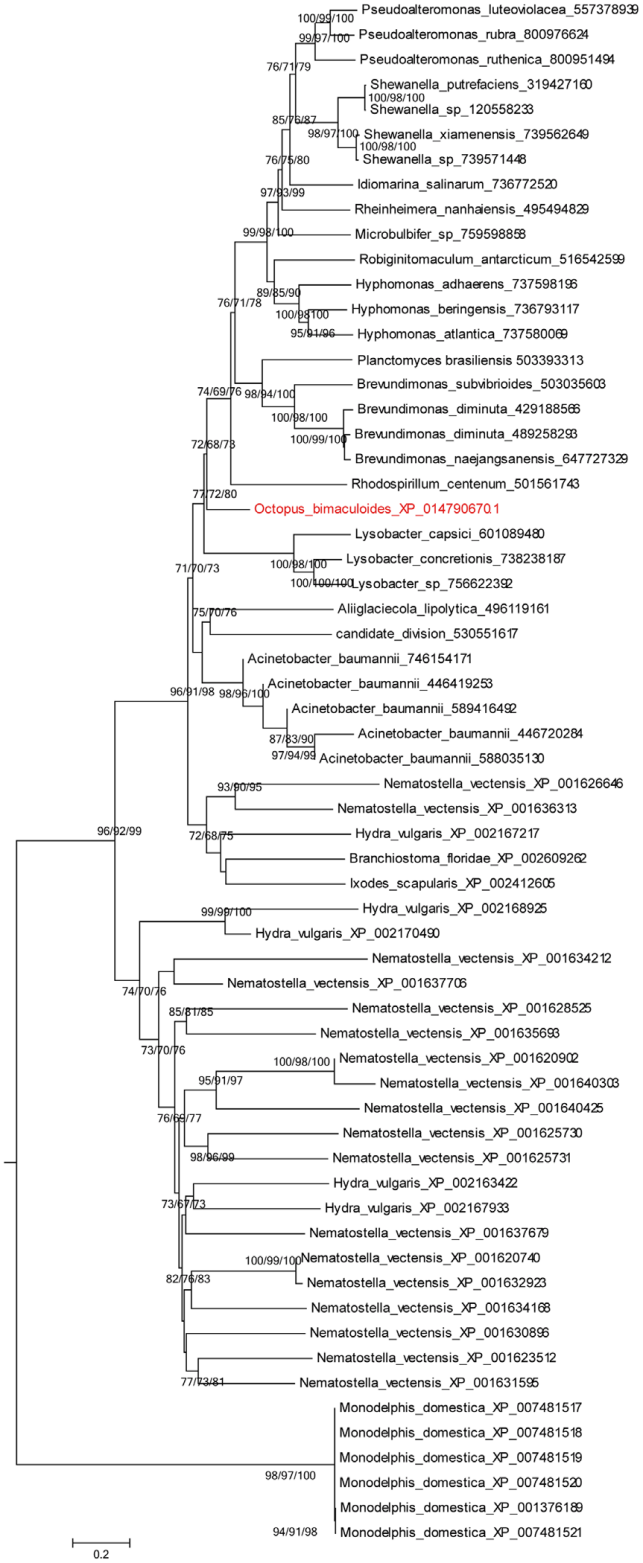
Phylogenetic analysis of the octopus UCP (XP_014790670.1) and their homologues.

Twelve HGT genes were validated as a result of three steps of BLAST search and two steps of phylogenetic analysis in 33,638 proteins of *O. bimaculoides*, which were aligned against the protein sequences of 2774 bacteria from NCBI (Supplementary Table 1), 26 protozoa, 50 fungi, 12 plants and 7 vertebrates from KEGG (Table 1). XP_014767445.1 (ZnMP1), XP_014774680.1 (ZnMP2), XP_014776931.1 (ZnMP3), XP_014776937.1 (ZnMP4), XP_014781458.1 (ZnMP5), XP_014782361.1 (ZnMP6), XP_014783435.1 (ZnMP7), XP_014779995.1 (ACS), XP_014783568.1 (D-AL), XP_014784751.1 (PGS), XP_014788506.1 (SRL) and XP_014790670.1 (UCP) were sifted out by the standard of phylogenetic incongruences, which were nested within bacteria homologs other than the vertebrate homologs. The phylogenetic trees were demonstrated in Fig. 1, Fig. 2, Fig. 3, Fig. 4, Fig. 5.

**Table 1:** Eukaryote genome sequences used in this study.

fugu (50)	plant (12)	vertebrate (7)	protozoa (26)
Zygosaccharomyces_rouxii	Zea mays (maize)	Xenopus (Silurana) tropicalis	Tetrahymena_thermophila
Yarrowia_lipolytica	Thalassiosira_pseudonana	Takifugu rubripes	Theileria_annulata
Vitis vinifera (wine grape)	Sorghum bicolor (sorghum)	Taeniopygia_guttata	Theileria_parva
Vanderwaltozyma_polyspora	Ricinus communis (castor bean)	Oryzias latipes	Toxoplasma_gondii
Ustilago_maydis	Populus trichocarpa (black cottonwood)	Gallus_gallus	Trichomonas_vaginalis
Uncinocarpus_reesii	Physcomitrella patens subsp. patens	Danio rerio	Trypanosoma_brucei
Sclerotinia_sclerotiorum	Phaeodactylum_tricornutum	Anolis carolinensis	Trypanosoma_cruzi
Saccharomyces_cerevisiae	Oryza brachyantha (malo sina)		Plasmodium_berghei
Saccharomyces mikatae	Oryza sativa japonica (Japanese rice)		Plasmodium_chabaudi
Saccharomyces paradoxus	Cyanidioschyzon merolae		plasmodium_falciparum_3d7
Scheffersomyces stipitis	Chlamydomonas reinhardtii		plasmodium_falciparum_dd2
Schizosaccharomyces pombe	Arabidopsis thaliana (thale cress)		plasmodium_falciparum_hb3
Postia_placenta			Plasmodium_knowlesi
Podospora_anserina			Plasmodium_vivax
Pichia guilliermondii			Plasmodium_yoelii
Phanerochaete_carnosa			Paramecium_tetraurelia
Phanerochaete chrysosporium			Monosiga_brevicollis
Penicillium_rubens			Leishmania_braziliensis
Ostreococcus lucimarinus			Leishmania_infantum
Ostreococcus tauri			Leishmania_major
Neosartorya_fischeri			Giardia_intestinalis
Neurospora_crassa			Entamoeba_dispar
Lodderomyces_elongisporus			Entamoeba_histolytica
Magnaporthe_oryzae			Encephalitozoon_cuniculi
Malassezia_globosa			Cryptosporidium_hominis
Moniliophthora_perniciosa			Cryptosporidium_parvum
Kluyveromyces_lactis			Babesia_bovis
Kluyveromyces waltii			
Komagataella pastoris GS115			
Laccaria_bicolor			
Lachancea_thermotolerans			
Fusarium_graminearum			
Dictyostelium_discoideum			
Debaryomyces_hansenii			
Cryptococcus_gattii			
Cryptococcus_neoformans			
Coccidioides_immitis			
Clavispora_lusitaniae			
Candida_albicans			
Candida_dubliniensis			
Candida_glabrata			
Candida_tropicalis			
Ashbya gossypii			
Aspergillus_clavatus			
Aspergillus_flavus			
Aspergillus_fumigatus			
Aspergillus_nidulans			
Aspergillus_niger			
Aspergillus_oryzae			
Botryotinia fuckeliana

CodonW was employed to investigate the codon usage bias among the HGT genes, conding genes in the genome of *O. bimaculoides* and donor bacteria. Supplementary Table 3 indicated the PCA details in the CodonW analysis.

To validate the existence and expression of the HGT genes, PCR assay was performed to clone the cDNA fragments of twelve sifted HGT genes, with the cDNA synthesised from the hepatopancreas mRNA of octopus used as a template and primers in Table 2. The specificity of PCR results was evaluated with agarose gel electrophoresis with ethidium bromide (EB) staining. Following this, after being extracted from agarose gel, the PCR products were sequenced to further validate the expression of the HGT genes (Table 3).

**Table 2:** Primers used in this study.

Gene name	Product Length (bp)	Primer sequence (5′–3′)
XP_014767445.1	248	F: TGCCTAATGAGGAAGAACAAGTC
		R: TGCCTAATGAGGAAGAACAAGTC
XP_014774680.1	544	F: ACGGATTCAGCACCAAGC
		R: CATTGCGGAAATAGCAGTCT
XP_014776931.1	291	F: AACCCGTCCTTAGACAAAGTG
		R: GCATGGTTTAATTCCCACAA
XP_014776937.1	316	F: ATATTGCGTGTTCATTACGGG
		R: TGTCCAAGGATGGGTCTGC
XP_014779995.1	263	F: CTTGACTGTCGTCCCTTGC
		R: GGATTCTGCTAAAAGCACCC
XP_014781458.1	293	F: AAACGCATTTTGGGATGG
		R: CAGATTCATTGTCTAAGGAGGG
XP_014782361.1	332	F: GGTTTGGCAAGCGGTTTT
		R: CCAGTGAGTAAATGGTCAGCAA
XP_014783435.1	260	F: CCAGTGAGTAAATGGTCAGCAA
		R: TTCTTCAGGGACCGCACTT
XP_014783568.1	274	F: GGCTGGAACTGAGAAAACCC
		R: CCAGTATGACCCCACGAACA
XP_014784751.1	489	F: CCTTGGATGTTGGTGGCA
		R: GCTTGGTTGGGATCGTTCTT
XP_014788506.1	509	F: ATCATTCAAGATACGCAACGC
		R: CCATACCGGAAGAAAGAGCA
XP_014790670.1	331	F: GTTCCCTTTCTACACGGCATT
		R: GGCTTTCTCAGCTTTCTTTCG

**Table 3:** Detailed nuclear sequences content of HGT genes validated by PCR.

ID	Sequence
XP_014767445.1	TGCCTAATGAGGAAGAACAACTCTATCACGTCAACTGCAAAGATGGTACGAAAGATGAAGCGAACGGAGCTTATTCTCCGATAAATGATGCTCTTTTCTATGGCAACATCATCTACAGTATGTGCATGGAATGGCTGAAAGCTCCTCCGAAAAAGGAGTTGCCTATGGTCTTCCGTGTGCACTATAGTAACGATGTGGTGAATGCTTTTTACAATGGCAGAAACTTTACGTTTGGTGATGGAGACTGG
XP_014774680.1	ACGGATTCGGCACCAAGCGTACAAGGAACAAACATCAAAAAGTACCACGAAACTTATCGAGGACTTCCAATGTTTGATGCCAGCCTCACTGTGGAGACGGACGCCAAAAGCCATGTGTACACTGGCCAGGTGACAGGTAAACTGGTTCAGAACCTTGATGATGACATCAACTCTACAATACCAAATGTGACTGAACAGGAAGCTGTACAATTGGCTCTGATGTATGGCAAATTTCCTATGGCAGGGGCCCGCATAGACAGCAGCAAACCTCAGTTGATAATCCATGTGAAGGACAATATCGGAATCCTGGTTTATCGAGTTCAATATTTCGCTGTGTCTGATGGAAAAAATTATCGATTTGGCATGATGATCAATGCAAAGAATGGAATGCTTGTAGACAAATGGAACACACTAGAAACTGCAAGAGAGAAACATCAGATGAAAGGTATAGGAGGCAACAAGTTGATAGTTAAAAAAACGTATGGGGAATTGCTACCTTATTTGCAAGTGAGACGCACTGGAGAAGACTGCTATTTCCGCAATG
XP_014776931.1	AACCCGTCCTTAGACAAAGTGTCTATAAGTCGTGTAGATAAATTCAATCAAGAAATGGATCCTCATCATGGAAGTGGGATTTATAATTTTATTTTTTACTACATTGTACACAATTTAAAAATGGATATCAAGGAGGCTTACCAAGTTTTCCTTATTGCCAATAAAATATATTGGCATCCTCATTCAGATTTCACTTCTGGGGCTTGTGATGTGTTGAAGGTTGCCTACGATCTTGGGAAAGATCTAGCCCCCTTTATTAAATCCTATGGGCTTGTGGGAATTAAACCATGC
XP_014776937.1	ATATTGCGTGTTCATTACGAGGAAAATTATGAAGATTCATACTGGAATGGGAAATATTGTACATTTGGTGATGGCCATACACGCTTTTATCCTTCAACAGACGCTGATGTTGTTGCACATGAATTTGCCCATGGTTTCACAGAACAACACTCTGGATTGATTTACTTTAACCAGTCTGGATCTATGAATGAAGCTTTTTCTGATATAACAGGGGAAGTCACTGAAGCTTACATAGATAAAAATGACTGGCTAATTGGCTTCTATATTGTGAAAGATAAGAAATCATACAGATTTATGACAGACCCATCCTTGGACA
XP_014779995.1	CTTGACTGTCGTCCCTTGCCCTGCTTCGTAGCTTACTTTTCCTTTCATATTTTTTGACCAAAAATAGTTAATGAAAAAGAATTGATAAAAATGTAAAAAAAAATATAACAATTTAAGTTGCTGGATTGACAAAACAGCAAAACTACAGACAGTATTCGTTCCGGCTTATTATTCTGAGTTCGAATCCCGCTGTCCTACTTGGCTACCAGACTTCACCTGTCTACCGGCTTAATACTATCACCGTGGGTGCTTTTAGCAGAATCC
XP_014781458.1	ATGAAAATTTTGGGATGGGGAATATTGCTCATTTGGTGATGGCGATACAAACGTTTATCCTCTTGTAGTTGCAGATGCAATTGGACATGAACTTGCTCATGGCTTCACAGAACAACACTCTGGATTGCTTTACAAAGACCAGTTTGGATCCATAAATGAGGCGTTTTCTGATATAACAGGAGAAGTCACTGAAGCTTATATGAGTGAGATTGACTGGTTTGTTGGCTTGGATGTCATTAAAGAAGAGGGTGCATTGAGATACATGGCAAACCCCTCCTTAGACAATGAATCTG
XP_014782361.1	GGTTTGGCAAGCGGTTTTGCTATCCACGGACTGAGTATCTATCATCACTGGGATATATTATTATCGGCGACATTAACCAAGGAAGAGGCTTTCGACATTACAATCATCTCGGCTGGGCACGGTCAGTTTAAAAAGTACATTTACAACTATAATTCGCACAGAAATGTTTACGTCGATGATTTCGGTGATGTAAGTTTGATCTACGAAATTGATTACCTAATATATACTGATGAAGTGGTTAAACGACCGGCGTTTCTTATAAGCGCTCATACCGGAGATATTTTGTTACAGTGGTCGAAGCTTGATACTTTTGCTGACCATTTACTCACTGG
XP_014783435.1	TAAGCAGACGGAAAGGATGGGGTACGAAAAAGGTTTTGAAGACTGCAGCTCATTCCAATCGTTTCTACTGGCATCCGTCAACTACTTTCGTTGAGGCCGCTTGTGACTTCATGAAGTCCGCTTATGACTCTGGATATGACACTAAACCTGTAGAGAGAGTTTTTAGAAAGGTTGGTATAGAAGTATGTCATCTGTCTTCATACATCCGAACAGTACACCAGAACTCGAAAATCGAGGGATTAAGTGCGGTCCCTGAAGAA
XP_014783568.1	GGCTGGAACTGAGAAAACCCCAAACTTTCCAAGGATGGACCCTTCCACATTCGAGTCTTGTACAAATGGATGGAGCGGAATCGTATGGAGGGTGGGTCGCCAACGCCAGTGACGTCCTCCAAATATTTGATTGTTTGTCTGAGAATCAGTGCAAATTGCTAGAAGGGGAGACCGTTCAGATGATGCTCAGCCGTCCTGAATATGAGAATGGTGACGAATGGTATGGATTTGGTTTAGAGGTAGATAATGGCTGTTCGTGGGGTCATACTGG
XP_014784751.1	CCTTGGATGTTGGTGGCAACTTGAACCGGGAATGACTTGGCAATGGCAGTTGACTAGTCCGCTGGATCTTACCTATGAAGTAGAGATGTATGATGTAGATCTATACGACACGTATGAACCGCAGTTCGACTACCTTAAAAAGTCACACATTCTGGTAGTCTGCTACATATCAGTCGGAACTTGGGAAAATTGGCGTCCAGATGCTCACAGTTTTCCAAACTCTACTCTAGGTAAACCTCTCTCTAAATGGCCAGGTGAGAGATGGCTAGATATTCGTAGTCCAGATGTAAAACGGATTATTTCGCAGGGAATCGAATTAGCTAGACGACGTGGTTGTGATGGTATTGAACCAGATAACATTTATGCATACGAAAATGACAACGGTTTGGGATTAACCGCTAATGACCAGCTCCAGTACAACATTTGGCTTTCTGTTGAGGCACATTCCCGTAATCTATCCATTGGTCTAAAGAACGATCCCAACCAAGC
XP_014788506.1	ATCATTCAAGATACGCAACGCTTTTTGTAGCTTCAAAAACCTTCCTCCGAATACCAAAGCTGTGTACACTGTAAGCACGGGTAACTTTGCTCGTGCCGTCGCATGGTTCGGCAGAAGAAACAACATCAAATGTACAATCCTAGTTCCAGATCATGCACCAAAATGCAAAACAGCCGCTGTTGAAAAGTTAGGTGCTGGCATAGTAAGGATTGAACATAAAGAATGGTTTGAAATCATAGCTGGTCGCAAGACTTAAGAGAAAGGAGAAAACCTTGGTGTTTACTTACATCCAGAATTTGATAGGAATGTTCTCGCAGGTATTTCCACATATCCTCTTTGTTGTTATTGTGGCTGCTGCTGTTGTTGTTGCTACTGTCACACTTATGTAATATCTTCTGCTTTCTTTTGTAAAGGAAACGGCACAGTTGCTTTGGAGATATTGCAAGAGTGACCGGACGTGGACACAATAATAATACCATATGGAGGAGGTGCTCTTTCTTCCGGTATGG
XP_014790670.1	GTTCCCTTTCTACACGGCATTTGTTGAGGGTTGGGGCCTATACTCGGAGTTCCTTGGAGAAGAAATGGGGATGTACAAAACCGATTATGACCGGATCGGTTCGTTTTGCATTTGAGCTTTTGCGAGCTCATCGTCTGTTCATTGATACAGGAATCCATGCAAAACAAATGACTCGGCAGCATGGAATCGATCTCCTGACCAACTTCACAGGCCTCAGTGAGAAACAAGCATCAATTGAGGTTGACCGCTACATTACCATCCCGGGTCAGGCCTGTGCTTATAAATTCGGAGAACTGAGGATTCTAGAACTGCGAAAGAAAGCTGAGAAAGCC

**Fig. 6:**
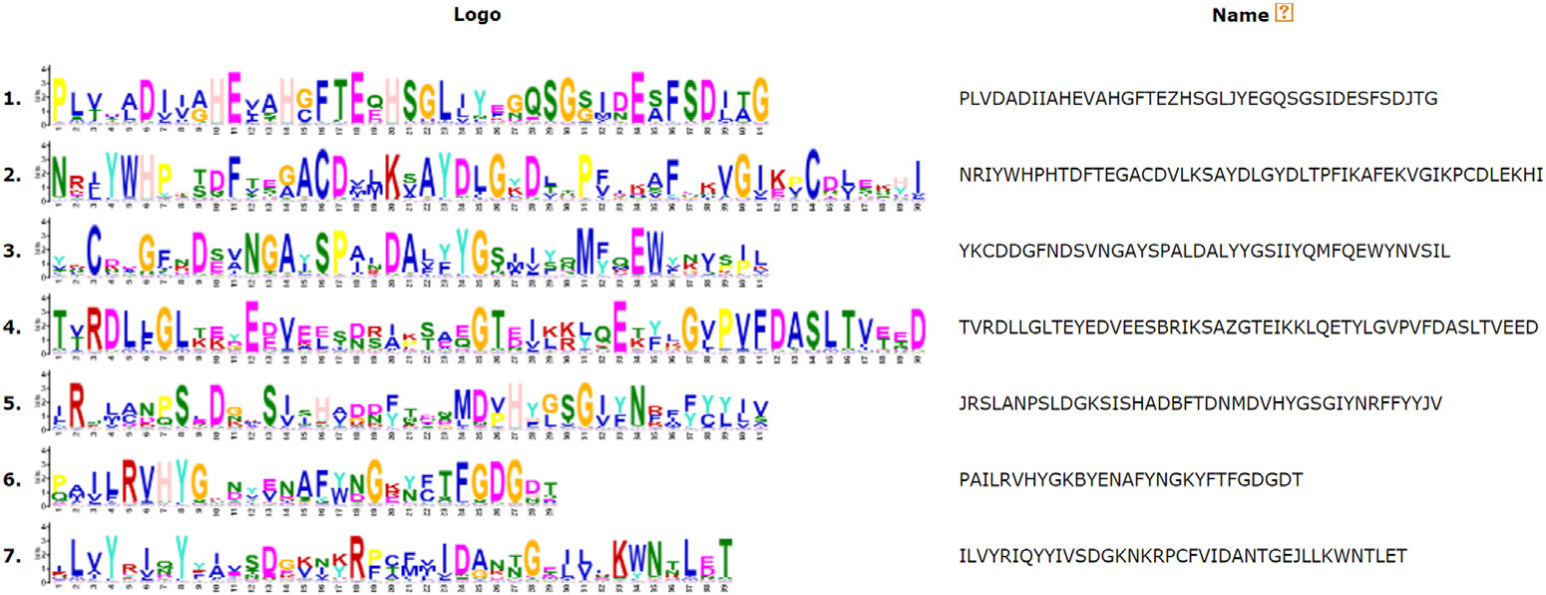
Detailed motif introduction of HGT ZnMPs.

Motif search was employed to compare the motif locations between the HGT and endogenous genes. In the HGT and endogenous Zn-metalloproteinases, 7 types of motif were detected (Fig. 6). The motifs were similar in proteins of the horizontally acquired Zn-metalloproteinases, but differed to endogenous proteins.

## 2 Experimental design, materials, and methods

### 2.1 Determination of HGT Based on BLAST Search and Phylogenetic Analysis

*O. bimaculoides* genome sequences were downloaded from the National Center for Biotechnology Information (NCBI, v2_0 version, GCA_001194135.1) and 33,638 protein-coding genes were employed in the present study [1]. The bacteria sequences of 2,774 species were also collected from the NCBI ftp site. Additionally, genome sequences of 26 protozoa, 50 fungi, 12 plants and 7 vertebrates were downloaded from the Kyoto encyclopedia of genes and genomes (KEGG, www.genome.jp/kegg/) database. It was composed by three rounds of BLAST alignment and two rounds of phylogenetic analysis. The HGT determination process was performed according to previous reports with modifications [2]. The BLASTP search was performed to detect similar protein sequences between *O. bimaculoides* and the local database constructed by bacteria with an E value ≤10^−30^, coverage value ≥25% and identity value ≥25%. Following this, the BLASTP program with the same threshold was employed to estimate the distribution spectrum of sifted similar genes in 26 protozoa, 50 fungi, 12 plants and 7 vertebrates. The candidate genes with similar genes from 2 or more species were rejected. Following this, the sifted genes were adopted to BLASTP research against NCBI non-redundant (NR) protein database with an E value ≤10^−3^, coverage value ≥30% and identity value ≥30%. Phylogenetic analysis was composed with two steps. We used MUSCLE 3.8.31 (http://www.drive5.com/muscle/) and FastTree (http://www.microbesonline.org/fasttree/) in the first step to construct a Maximum likelihood (ML) tree. After this, CLUSTALX 2.0 (http://www.clustal.org) and MEGA 7.0 (http://www.mega.co.nz) were used based on genes selected in the first step for the NJ and ML trees reconstruction. The phylogenetic trees were select based on the phylogenetic topology patterns reported by Stanhope [3]. After the second tree construction analysis, octopus genes with explicit topologies of HGT type were considered as the candidate sequences.

### 2.2 Detection of codon usage bias

The correspondence analysis of codon usage bias was carried out to measure the degree of adaptation in the octopus HGT genes and the predicted bacteria donors. Codon usage analysis was performed using CodonW (http://codonw.sourceforge.net), and a primary orthogonal axis representing the greatest variation within the data was employed in the correspondence analysis.

### 2.3 PCR validation of HGT genes

Adult octopuses were collected from a local market in Shenzhen, Guangdong Province, China, and maintained in aerated fresh seawater at 20±2 °C for a week before processing. Before sampling, the octopuses were washed by the sterile sea water and incubated in 75% alcohol for 1 min. Total RNA was isolated from octopus hepatopancreas using Trizol reagent (TaKaRa) following its protocol. The first strand cDNA synthesis was carried out based on Promega M -MLV RT Usage information using the DNase I (Promega)-treated total RNA as a template and oligo (dT)-adaptor as the primer. The reaction was performed at 42 °C for 1 h, terminated by heating at 95 °C for 5 min. The cDNA sequence fragments of HGT genes were cloned by PCR with primers. Following this, after detection by agarose gel electrophoresis, the PCR products were sequenced.

### 2.4 Zn-metalloproteinase family analysis

By Multiple EM for the Motif Elicitation (MEME) suite, the conserved motifs of the gene family were analyzed [4].
