# Supplementary material for: Data on horizontally transferred genes in California two-spot octopus, Octopus bimaculoides
Source: Data Brief. 2018 Jun 1;19:1274–86. doi: 10.1016/j.dib.2018.05.132 (PMC6011040; doi:10.1016/j.dib.2018.05.132)
Supplement: Supplementary file 1 — Supplementary material [file mmc1.docx]

Conflicts of Interest

The authors declare no conflict of interest.
