# Supplementary material for: Data on horizontally transferred genes in California two-spot octopus, Octopus bimaculoides
Source: Data Brief. 2018 Jun 1;19:1274–86. doi: 10.1016/j.dib.2018.05.132 (PMC6011040; doi:10.1016/j.dib.2018.05.132)
Supplement: Supplementary file 2 — Supplementary material [file mmc2.docx]

Table 1 Bacteria genome sequences used in this study.

| Acaryochloris_marina_MBIC11017_uid58167  Acetobacter_pasteurianus_386B_uid214433  Acetobacter_pasteurianus_IFO_3283_01_42C_uid158377  Acetobacter_pasteurianus_IFO_3283_01_uid59279  Acetobacter_pasteurianus_IFO_3283_03_uid158373  Acetobacter_pasteurianus_IFO_3283_07_uid158381  Acetobacter_pasteurianus_IFO_3283_12_uid158379  Acetobacter_pasteurianus_IFO_3283_22_uid158383  Acetobacter_pasteurianus_IFO_3283_26_uid158531  Acetobacter_pasteurianus_IFO_3283_32_uid158375  Acetobacterium_woodii_DSM_1030_uid88073  Acetohalobium_arabaticum_DSM_5501_uid51423  Acholeplasma_brassicae_uid222823  Acholeplasma_laidlawii_PG_8A_uid58901  Acholeplasma_palmae_J233_uid222824  Achromobacter_xylosoxidans_A8_uid59899  Achromobacter_xylosoxidans_NBRC_15126_uid232243  Achromobacter_xylosoxidans_uid205255  Acidaminococcus_fermentans_DSM_20731_uid43471  Acidaminococcus_intestini_RyC_MR95_uid74445  Acidianus_hospitalis_W1_uid66875  Acidilobus_saccharovorans_345_15_uid51395  Acidimicrobidae_bacterium_YM16_304_uid193703  Acidimicrobium_ferrooxidans_DSM_10331_uid59215  Acidiphilium_cryptum_JF_5_uid58447  Acidiphilium_multivorum_AIU301_uid63345  Acidithiobacillus_caldus_SM_1_uid70791  Acidithiobacillus_ferrivorans_SS3_uid67387  Acidithiobacillus_ferrooxidans_ATCC_23270_uid57649  Acidithiobacillus_ferrooxidans_ATCC_53993_uid58613  Acidobacterium_MP5ACTX9_uid50551  Acidobacterium_capsulatum_ATCC_51196_uid59127  Acidothermus_cellulolyticus_11B_uid58501  Acidovorax_JS42_uid58427  Acidovorax_KKS102_uid176500  Acidovorax_avenae_ATCC_19860_uid42497  Acidovorax_citrulli_AAC00_1_uid58429  Acidovorax_ebreus_TPSY_uid59233  Aciduliprofundum_MAR08_339_uid184407  Aciduliprofundum_boonei_T469_uid43333  Acinetobacter_ADP1_uid61597  Acinetobacter_baumannii_1656_2_uid158677  Acinetobacter_baumannii_AB0057_uid59083  Acinetobacter_baumannii_AB307_0294_uid59271  Acinetobacter_baumannii_ACICU_uid58765  Acinetobacter_baumannii_ATCC_17978_uid58731  Acinetobacter_baumannii_AYE_uid61637  Acinetobacter_baumannii_BJAB07104_uid210971  Acinetobacter_baumannii_BJAB0715_uid210972  Acinetobacter_baumannii_BJAB0868_uid210973  Acinetobacter_baumannii_D1279779_uid190222  Acinetobacter_baumannii_MDR_TJ_uid162739  Acinetobacter_baumannii_MDR_ZJ06_uid158685  Acinetobacter_baumannii_SDF_uid61601  Acinetobacter_baumannii_TCDC_AB0715_uid158679  Acinetobacter_baumannii_TYTH_1_uid176498  Acinetobacter_baumannii_ZW85_1_uid231518  Acinetobacter_calcoaceticus_PHEA_2_uid83123  Acinetobacter_oleivorans_DR1_uid50119  Actinobacillus_pleuropneumoniae_serovar_3_JL03_uid58891  Actinobacillus_pleuropneumoniae_serovar_5b_L20_uid58789  Actinobacillus_pleuropneumoniae_serovar_7_AP76_uid59231  Actinobacillus_succinogenes_130Z_uid58247  Actinobacillus_suis_H91_0380_uid176363  Actinoplanes_N902_109_uid202219  Actinoplanes_SE50_110_uid162333  Actinoplanes_friuliensis_DSM_7358_uid226110  Actinoplanes_missouriensis_431_uid158169  Actinosynnema_mirum_DSM_43827_uid58951  Adlercreutzia_equolifaciens_DSM_19450_uid223286  Advenella_kashmirensis_WT001_uid80859  Aequorivita_sublithincola_DSM_14238_uid168181  Aerococcus_urinae_ACS_120_V_Col10a_uid64757  Aeromonas_hydrophila_ATCC_7966_uid58617  Aeromonas_hydrophila_ML09_119_uid205540  Aeromonas_salmonicida_A449_uid58631  Aeromonas_veronii_B565_uid66323  Aeropyrum_camini_SY1___JCM_12091_uid222311  Aeropyrum_pernix_K1_uid57757  Aggregatibacter_actinomycetemcomitans_ANH9381_uid80743  Aggregatibacter_actinomycetemcomitans_D11S_1_uid41333  Aggregatibacter_actinomycetemcomitans_D7S_1_uid46989  Aggregatibacter_aphrophilus_NJ8700_uid59407  Agrobacterium_H13_3_uid63403  Agrobacterium_fabrum_C58_uid57865  Agrobacterium_radiobacter_K84_uid58269  Agrobacterium_vitis_S4_uid58249  Agromonas_oligotrophica_S58_uid192186  Akkermansia_muciniphila_ATCC_BAA_835_uid58985  Alcanivorax_borkumensis_SK2_uid58169  Alcanivorax_dieselolei_B5_uid176364  Alicycliphilus_denitrificans_BC_uid49953  Alicycliphilus_denitrificans_K601_uid66307  Alicyclobacillus_acidocaldarius_DSM_446_uid59199  Alicyclobacillus_acidocaldarius_Tc_4_1_uid158681  Aliivibrio_salmonicida_LFI1238_uid59251  Alistipes_finegoldii_DSM_17242_uid168180  Alistipes_shahii_WAL_8301_uid197175  Alkalilimnicola_ehrlichii_MLHE_1_uid58467  Alkaliphilus_metalliredigens_QYMF_uid58171  Alkaliphilus_oremlandii_OhILAs_uid58495  Allochromatium_vinosum_DSM_180_uid46083  Alteromonas_SN2_uid67349  Alteromonas_macleodii_ATCC_27126_uid55253  Alteromonas_macleodii_AltDE1_uid179068  Alteromonas_macleodii__Aegean_Sea_MED64__uid231689  Alteromonas_macleodii__Balearic_Sea_AD45__uid176366  Alteromonas_macleodii__Black_Sea_11__uid176365  Alteromonas_macleodii__Deep_ecotype__uid58251  Alteromonas_macleodii__English_Channel_615__uid210781  Alteromonas_macleodii__English_Channel_673__uid176367  Alteromonas_macleodii__Ionian_Sea_U4__uid210780  Alteromonas_macleodii__Ionian_Sea_U7__uid210785  Alteromonas_macleodii__Ionian_Sea_U8__uid210782  Alteromonas_macleodii__Ionian_Sea_UM4b__uid210784  Alteromonas_macleodii__Ionian_Sea_UM7__uid210783  Aminobacterium_colombiense_DSM_12261_uid47083  Ammonifex_degensii_KC4_uid41053  Amphibacillus_xylanus_NBRC_15112_uid176453  Amycolatopsis_mediterranei_RB_uid216089  Amycolatopsis_mediterranei_S699_uid158689  Amycolatopsis_mediterranei_S699_uid171830  Amycolatopsis_mediterranei_U32_uid50565  Amycolatopsis_orientalis_HCCB10007_uid203791  Amycolicicoccus_subflavus_DQS3_9A1_uid67253  Anabaena_90_uid179383  Anabaena_cylindrica_PCC_7122_uid183339  Anabaena_variabilis_ATCC_29413_uid58043  Anaerobaculum_mobile_DSM_13181_uid168323  Anaerococcus_prevotii_DSM_20548_uid59219  Anaerolinea_thermophila_UNI_1_uid62245  Anaeromyxobacter_Fw109_5_uid58755  Anaeromyxobacter_K_uid58953  Anaeromyxobacter_dehalogenans_2CP_1_uid58989  Anaeromyxobacter_dehalogenans_2CP_C_uid58135  Anaplasma_centrale_Israel_uid42155  Anaplasma_marginale_Dawn_uid226994  Anaplasma_marginale_Florida_uid58577  Anaplasma_marginale_Gypsy_Plains_uid227217  Anaplasma_marginale_Maries_uid57629  Anaplasma_phagocytophilum_Dog2_uid213225  Anaplasma_phagocytophilum_HZ2_uid213224  Anaplasma_phagocytophilum_HZ_uid57951  Anaplasma_phagocytophilum_JM_uid213223  Anoxybacillus_flavithermus_WK1_uid59135  Aquifex_aeolicus_VF5_uid57765  Arcanobacterium_haemolyticum_DSM_20595_uid49489  Archaeoglobus_fulgidus_DSM_4304_uid57717  Archaeoglobus_profundus_DSM_5631_uid43493  Archaeoglobus_sulfaticallidus_PM70_1_uid201033  Archaeoglobus_veneficus_SNP6_uid65269  Arcobacter_L_uid158135  Arcobacter_butzleri_7h1h_uid200766  Arcobacter_butzleri_ED_1_uid158699  Arcobacter_butzleri_RM4018_uid58557  Arcobacter_nitrofigilis_DSM_7299_uid49001  Aromatoleum_aromaticum_EbN1_uid58231  Arthrobacter_FB24_uid58141  Arthrobacter_Rue61a_uid174511  Arthrobacter_arilaitensis_Re117_uid53509  Arthrobacter_aurescens_TC1_uid58109  Arthrobacter_chlorophenolicus_A6_uid58969  Arthrobacter_phenanthrenivorans_Sphe3_uid63629  Arthrospira_platensis_NIES_39_uid197171  Aster_yellows_witches_broom_phytoplasma_AYWB_uid58297  Asticcacaulis_excentricus_CB_48_uid55641  Atopobium_parvulum_DSM_20469_uid59195  Azoarcus_BH72_uid61603  Azoarcus_KH32C_uid193704  Azorhizobium_caulinodans_ORS_571_uid58905  Azospirillum_B510_uid46085  Azospirillum_brasilense_Sp245_uid162161  Azospirillum_lipoferum_4B_uid82343  Azotobacter_vinelandii_CA6_uid198830  Azotobacter_vinelandii_CA_uid198829  Azotobacter_vinelandii_DJ_uid57597  Bacillus_1NLA3E_uid81841  Bacillus_JS_uid162189  Bacillus_amyloliquefaciens_CC178_uid226115  Bacillus_amyloliquefaciens_DSM_7_uid53535  Bacillus_amyloliquefaciens_FZB42_uid58271  Bacillus_amyloliquefaciens_IT_45_uid181617  Bacillus_amyloliquefaciens_LFB112_uid232246  Bacillus_amyloliquefaciens_LL3_uid158133  Bacillus_amyloliquefaciens_TA208_uid158701  Bacillus_amyloliquefaciens_XH7_uid158881  Bacillus_amyloliquefaciens_Y2_uid165195  Bacillus_amyloliquefaciens_plantarum_AS43_3_uid183682  Bacillus_amyloliquefaciens_plantarum_CAU_B946_uid84215  Bacillus_amyloliquefaciens_plantarum_NAU_B3_uid222816  Bacillus_amyloliquefaciens_plantarum_UCMB5033_uid215237  Bacillus_amyloliquefaciens_plantarum_UCMB5036_uid190705  Bacillus_amyloliquefaciens_plantarum_UCMB5113_uid215236  Bacillus_amyloliquefaciens_plantarum_YAU_B9601_Y2_uid159001  Bacillus_anthracis_A0248_uid59385  Bacillus_anthracis_A2012_uid54101  Bacillus_anthracis_Ames_uid57909  Bacillus_anthracis_CDC_684_uid59303  Bacillus_anthracis_H9401_uid162021  Bacillus_anthracis_Sterne_uid58091  Bacillus_anthracis__Ames_Ancestor__uid58083  Bacillus_atrophaeus_1942_uid59887  Bacillus_cellulosilyticus_DSM_2522_uid43329  Bacillus_cereus_03BB102_uid59299  Bacillus_cereus_AH187_uid58753  Bacillus_cereus_AH820_uid58751  Bacillus_cereus_ATCC_10987_uid57673  Bacillus_cereus_ATCC_14579_uid57975  Bacillus_cereus_B4264_uid58757  Bacillus_cereus_E33L_uid58103  Bacillus_cereus_F837_76_uid83611  Bacillus_cereus_FRI_35_uid173403  Bacillus_cereus_G9842_uid58759  Bacillus_cereus_NC7401_uid82815  Bacillus_cereus_Q1_uid58529  Bacillus_cereus_biovar_anthracis_CI_uid50615  Bacillus_clausii_KSM_K16_uid58237  Bacillus_coagulans_2_6_uid68053  Bacillus_coagulans_36D1_uid54335  Bacillus_cytotoxicus_NVH_391_98_uid58317  Bacillus_halodurans_C_125_uid57791  Bacillus_infantis_NRRL_B_14911_uid222804  Bacillus_licheniformis_9945A_uid207072  Bacillus_licheniformis_ATCC_14580_uid58097  Bacillus_licheniformis_DSM_13___ATCC_14580_uid58199  Bacillus_megaterium_DSM319_uid48371  Bacillus_megaterium_QM_B1551_uid15862  Bacillus_megaterium_WSH_002_uid159841  Bacillus_pseudofirmus_OF4_uid45847  Bacillus_pumilus_SAFR_032_uid59017  Bacillus_selenitireducens_MLS10_uid49513  Bacillus_subtilis_168_uid57675  Bacillus_subtilis_6051_HGW_uid193706  Bacillus_subtilis_BAB_1_uid195461  Bacillus_subtilis_BSP1_uid184010  Bacillus_subtilis_BSn5_uid62463  Bacillus_subtilis_PY79_uid229877  Bacillus_subtilis_QB928_uid173926  Bacillus_subtilis_RO_NN_1_uid158879  Bacillus_subtilis_XF_1_uid189187  Bacillus_subtilis_natto_BEST195_uid183001  Bacillus_subtilis_spizizenii_TU_B_10_uid73967  Bacillus_subtilis_spizizenii_W23_uid51879  Bacillus_thuringiensis_Al_Hakam_uid58795  Bacillus_thuringiensis_BMB171_uid49135  Bacillus_thuringiensis_Bt407_uid177931  Bacillus_thuringiensis_HD_771_uid173374  Bacillus_thuringiensis_HD_789_uid173860  Bacillus_thuringiensis_MC28_uid176369  Bacillus_thuringiensis_YBT_1518_uid229419  Bacillus_thuringiensis_serovar_IS5056_uid190186  Bacillus_thuringiensis_serovar_chinensis_CT_43_uid158151  Bacillus_thuringiensis_serovar_finitimus_YBT_020_uid158875  Bacillus_thuringiensis_serovar_konkukian_97_27_uid58089  Bacillus_thuringiensis_serovar_kurstaki_HD73_uid189188  Bacillus_toyonensis_BCT_7112_uid227218  Bacillus_weihenstephanensis_KBAB4_uid58315  Bacteriovorax_marinus_SJ_uid82341  Bacteroides_CF50_uid222805  Bacteroides_fragilis_638R_uid84217  Bacteroides_fragilis_NCTC_9343_uid57639  Bacteroides_fragilis_YCH46_uid58195  Bacteroides_helcogenes_P_36_108_uid62135  Bacteroides_salanitronis_DSM_18170_uid63269  Bacteroides_thetaiotaomicron_VPI_5482_uid62913  Bacteroides_uniformis_uid13130  Bacteroides_vulgatus_ATCC_8482_uid58253  Bacteroides_xylanisolvens_XB1A_uid197168  Bartonella_australis_Aust_NH1_uid189950  Bartonella_bacilliformis_KC583_uid58533  Bartonella_clarridgeiae_73_uid62131  Bartonella_grahamii_as4aup_uid59405  Bartonella_henselae_Houston_1_uid57745  Bartonella_quintana_RM_11_uid174512  Bartonella_quintana_Toulouse_uid57635  Bartonella_tribocorum_CIP_105476_uid59129  Bartonella_vinsonii_berkhoffii_Winnie_uid189951  Baumannia_cicadellinicola_Hc__Homalodisca_coagulata__uid58111  Bdellovibrio_bacteriovorus_HD100_uid61595  Bdellovibrio_bacteriovorus_Tiberius_uid182482  Bdellovibrio_exovorus_JSS_uid194119  Beijerinckia_indica_ATCC_9039_uid59057  Belliella_baltica_DSM_15883_uid168182  Beutenbergia_cavernae_DSM_12333_uid59047  Bibersteinia_trehalosi_192_uid193709  Bifidobacterium_adolescentis_ATCC_15703_uid58559  Bifidobacterium_animalis_ATCC_25527_uid162513  Bifidobacterium_animalis_lactis_AD011_uid58911  Bifidobacterium_animalis_lactis_ATCC_27673_uid222803  Bifidobacterium_animalis_lactis_B420_uid163691  Bifidobacterium_animalis_lactis_BB_12_uid158871  Bifidobacterium_animalis_lactis_BLC1_uid158867  Bifidobacterium_animalis_lactis_Bi_07_uid163693  Bifidobacterium_animalis_lactis_Bl12_uid210081  Bifidobacterium_animalis_lactis_Bl_04_uid59359  Bifidobacterium_animalis_lactis_CNCM_I_2494_uid158869  Bifidobacterium_animalis_lactis_DSM_10140_uid59357  Bifidobacterium_animalis_lactis_V9_uid158865  Bifidobacterium_asteroides_PRL2011_uid176921  Bifidobacterium_bifidum_BGN4_uid167988  Bifidobacterium_bifidum_PRL2010_uid59883  Bifidobacterium_bifidum_S17_uid59545  Bifidobacterium_breve_ACS_071_V_Sch8b_uid158863  Bifidobacterium_breve_UCC2003_uid193702  Bifidobacterium_dentium_Bd1_uid43091  Bifidobacterium_longum_BBMN68_uid60163  Bifidobacterium_longum_DJO10A_uid58833  Bifidobacterium_longum_F8_uid197184  Bifidobacterium_longum_JCM_1217_uid62695  Bifidobacterium_longum_JDM301_uid49131  Bifidobacterium_longum_KACC_91563_uid158861  Bifidobacterium_longum_NCC2705_uid57939  Bifidobacterium_longum_infantis_157F_uid62693  Bifidobacterium_longum_infantis_ATCC_15697_uid159865  Bifidobacterium_longum_infantis_ATCC_15697_uid58677  Bifidobacterium_thermophilum_RBL67_uid193770  Blastococcus_saxobsidens_DD2_uid89391  Blattabacterium__Blaberus_giganteus__uid165873  Blattabacterium__Blatta_orientalis__Tarazona_uid188115  Blattabacterium__Blattella_germanica__Bge_uid41533  Blattabacterium__Cryptocercus_punctulatus__Cpu_uid81083  Blattabacterium__Mastotermes_darwiniensis__MADAR_uid77127  Blattabacterium__Nauphoeta_cinerea__uid222815  Blattabacterium__Panesthia_angustipennis_spadica__BPAA_uid193717  Blattabacterium__Periplaneta_americana__BPLAN_uid41287  Bordetella_avium_197N_uid61563  Bordetella_bronchiseptica_253_uid178913  Bordetella_bronchiseptica_MO149_uid177517  Bordetella_bronchiseptica_RB50_uid57613  Bordetella_parapertussis_12822_uid57615  Bordetella_parapertussis_18323_uid175569  Bordetella_parapertussis_Bpp5_uid177516  Bordetella_pertussis_CS_uid158859  Bordetella_pertussis_Tohama_I_uid57617  Bordetella_petrii_uid61631  Borrelia_afzelii_HLJ01_uid177930  Borrelia_afzelii_PKo_uid159867  Borrelia_afzelii_PKo_uid58653  Borrelia_bissettii_DN127_uid71231  Borrelia_burgdorferi_B31_uid57581  Borrelia_burgdorferi_CA382_uid214794  Borrelia_burgdorferi_JD1_uid161197  Borrelia_burgdorferi_N40_uid161241  Borrelia_burgdorferi_ZS7_uid59429  Borrelia_crocidurae_Achema_uid162335  Borrelia_duttonii_Ly_uid58791  Borrelia_garinii_BgVir_uid162165  Borrelia_garinii_NMJW1_uid177081  Borrelia_garinii_PBi_uid58125  Borrelia_hermsii_DAH_uid59225  Borrelia_miyamotoi_LB_2001_uid215233  Borrelia_recurrentis_A1_uid58793  Borrelia_turicatae_91E135_uid58311  Borrelia_valaisiana_VS116_uid54823  Brachybacterium_faecium_DSM_4810_uid58649  Brachyspira_hyodysenteriae_WA1_uid59291  Brachyspira_intermedia_PWS_A_uid158369  Brachyspira_murdochii_DSM_12563_uid48819  Brachyspira_pilosicoli_95_1000_uid50609  Brachyspira_pilosicoli_B2904_uid175255  Brachyspira_pilosicoli_P43_6_78_uid184077  Brachyspira_pilosicoli_WesB_uid175256  Bradyrhizobium_BTAi1_uid58505  Bradyrhizobium_ORS_278_uid58941  Bradyrhizobium_S23321_uid158167  Bradyrhizobium_japonicum_USDA_110_uid57599  Bradyrhizobium_japonicum_USDA_6_uid158851  Brevibacillus_brevis_NBRC_100599_uid59175  Brevundimonas_subvibrioides_ATCC_15264_uid42117  Brucella_abortus_A13334_uid83615  Brucella_abortus_S19_uid58873  Brucella_abortus_bv__1_9_941_uid58019  Brucella_canis_ATCC_23365_uid59009  Brucella_canis_HSK_A52141_uid83613  Brucella_ceti_TE10759_12_uid229880  Brucella_ceti_TE28753_12_uid229879  Brucella_melitensis_ATCC_23457_uid59241  Brucella_melitensis_M28_uid158857  Brucella_melitensis_M5_90_uid158855  Brucella_melitensis_NI_uid158853  Brucella_melitensis_biovar_Abortus_2308_uid62937  Brucella_melitensis_bv__1_16M_uid57735  Brucella_microti_CCM_4915_uid59319  Brucella_ovis_ATCC_25840_uid58113  Brucella_pinnipedialis_B2_94_uid71131  Brucella_suis_1330_uid159871  Brucella_suis_1330_uid57927  Brucella_suis_ATCC_23445_uid59015  Brucella_suis_VBI22_uid83617  Buchnera_aphidicola_5A__Acyrthosiphon_pisum__uid59285  Buchnera_aphidicola_APS__Acyrthosiphon_pisum__uid57805  Buchnera_aphidicola_Ak__Acyrthosiphon_kondoi__uid158533  Buchnera_aphidicola_Bp__Baizongia_pistaciae__uid57827  Buchnera_aphidicola_Cc__Cinara_cedri__uid58579  Buchnera_aphidicola_JF98__Acyrthosiphon_pisum__uid158845  Buchnera_aphidicola_JF99__Acyrthosiphon_pisum__uid158847  Buchnera_aphidicola_LL01__Acyrthosiphon_pisum__uid158843  Buchnera_aphidicola_Sg__Schizaphis_graminum__uid57913  Buchnera_aphidicola_TLW03__Acyrthosiphon_pisum__uid158849  Buchnera_aphidicola_Tuc7__Acyrthosiphon_pisum__uid59283  Buchnera_aphidicola_Ua__Uroleucon_ambrosiae__uid158535  Buchnera_aphidicola__Cinara_tujafilina__uid68101  Burkholderia_383_uid58073  Burkholderia_CCGE1001_uid42975  Burkholderia_CCGE1002_uid42523  Burkholderia_CCGE1003_uid46253  Burkholderia_KJ006_uid165871  Burkholderia_RPE64_uid205541  Burkholderia_YI23_uid81081  Burkholderia_ambifaria_AMMD_uid58303  Burkholderia_ambifaria_MC40_6_uid58701  Burkholderia_cenocepacia_AU_1054_uid58371  Burkholderia_cenocepacia_HI2424_uid58369  Burkholderia_cenocepacia_J2315_uid57953  Burkholderia_cenocepacia_MC0_3_uid58769  Burkholderia_cepacia_GG4_uid173858  Burkholderia_gladioli_BSR3_uid66301  Burkholderia_glumae_BGR1_uid59397  Burkholderia_mallei_ATCC_23344_uid57725  Burkholderia_mallei_NCTC_10229_uid58383  Burkholderia_mallei_NCTC_10247_uid58385  Burkholderia_mallei_SAVP1_uid58387  Burkholderia_multivorans_ATCC_17616_uid58697  Burkholderia_multivorans_ATCC_17616_uid58909  Burkholderia_phenoliruptrix_BR3459a_uid176370  Burkholderia_phymatum_STM815_uid58699  Burkholderia_phytofirmans_PsJN_uid58729  Burkholderia_pseudomallei_1026b_uid162511  Burkholderia_pseudomallei_1106a_uid58515  Burkholderia_pseudomallei_1710b_uid58391  Burkholderia_pseudomallei_668_uid58389  Burkholderia_pseudomallei_BPC006_uid174460  Burkholderia_pseudomallei_K96243_uid57733  Burkholderia_pseudomallei_MSHR305_uid213227  Burkholderia_pseudomallei_MSHR346_uid55259  Burkholderia_pseudomallei_NCTC_13179_uid226109  Burkholderia_rhizoxinica_HKI_454_uid60487  Burkholderia_thailandensis_E264_uid58081  Burkholderia_thailandensis_MSMB121_uid201037  Burkholderia_vietnamiensis_G4_uid58075  Burkholderia_xenovorans_LB400_uid57823  Butyrivibrio_fibrisolvens_uid197155  Butyrivibrio_proteoclasticus_B316_uid51489  Caldicellulosiruptor_bescii_DSM_6725_uid59201  Caldicellulosiruptor_hydrothermalis_108_uid60157  Caldicellulosiruptor_kristjanssonii_177R1B_uid60393  Caldicellulosiruptor_kronotskyensis_2002_uid60491  Caldicellulosiruptor_lactoaceticus_6A_uid60575  Caldicellulosiruptor_obsidiansis_OB47_uid51501  Caldicellulosiruptor_owensensis_OL_uid60165  Caldicellulosiruptor_saccharolyticus_DSM_8903_uid58289  Caldilinea_aerophila_DSM_14535___NBRC_104270_uid158165  Caldisericum_exile_AZM16c01_uid158173  Caldisphaera_lagunensis_DSM_15908_uid183486  Calditerrivibrio_nitroreducens_DSM_19672_uid60821  Caldivirga_maquilingensis_IC_167_uid58711  Calothrix_PCC_6303_uid183109  Calothrix_PCC_7507_uid182930  Campylobacter_03_427_uid226993  Campylobacter_coli_15_537360_uid226113  Campylobacter_coli_76339_uid217050  Campylobacter_coli_CVM_N29710_uid219322  Campylobacter_concisus_13826_uid58667  Campylobacter_curvus_525_92_uid58669  Campylobacter_fetus_82_40_uid58545  Campylobacter_hominis_ATCC_BAA_381_uid58981  Campylobacter_jejuni_00_2425_uid219359  Campylobacter_jejuni_00_2426_uid219324  Campylobacter_jejuni_00_2538_uid219325  Campylobacter_jejuni_00_2544_uid219326  Campylobacter_jejuni_4031_uid222817  Campylobacter_jejuni_81116_uid58771  Campylobacter_jejuni_81_176_uid58503  Campylobacter_jejuni_IA3902_uid159531  Campylobacter_jejuni_ICDCCJ07001_uid61249  Campylobacter_jejuni_M1_uid159535  Campylobacter_jejuni_NCTC_11168_BN148_uid174152  Campylobacter_jejuni_NCTC_11168___ATCC_700819_uid57587  Campylobacter_jejuni_PT14_uid176499  Campylobacter_jejuni_RM1221_uid57899  Campylobacter_jejuni_S3_uid159533  Campylobacter_jejuni_doylei_269_97_uid58671  Campylobacter_lari_RM2100_uid58115  Candidatus_Accumulibacter_phosphatis_clade_IIA_UW_1_uid59207  Candidatus_Amoebophilus_asiaticus_5a2_uid58963  Candidatus_Arthromitus_SFB_mouse_Japan_uid71379  Candidatus_Arthromitus_SFB_mouse_Yit_uid159517  Candidatus_Arthromitus_SFB_rat_Yit_uid73425  Candidatus_Azobacteroides_pseudotrichonymphae_genomovar__CFP2_uid59163  Candidatus_Blochmannia_chromaiodes_640_uid185308  Candidatus_Blochmannia_floridanus_uid57999  Candidatus_Blochmannia_pennsylvanicus_BPEN_uid58329  Candidatus_Blochmannia_vafer_BVAF_uid62083  Candidatus_Caldiarchaeum_subterraneum_uid227223  Candidatus_Carsonella_ruddii_CE_isolate_Thao2000_uid172732  Candidatus_Carsonella_ruddii_CS_isolate_Thao2000_uid172733  Candidatus_Carsonella_ruddii_DC_uid213383  Candidatus_Carsonella_ruddii_HC_isolate_Thao2000_uid172734  Candidatus_Carsonella_ruddii_HT_isolate_Thao2000_uid172735  Candidatus_Carsonella_ruddii_PC_isolate_NHV_uid172736  Candidatus_Carsonella_ruddii_uid58773  Candidatus_Chloracidobacterium_thermophilum_B_uid73587  Candidatus_Cloacamonas_acidaminovorans_Evry_uid62959  Candidatus_Desulforudis_audaxviator_MP104C_uid59067  Candidatus_Hamiltonella_defensa_5AT__Acyrthosiphon_pisum__uid59289  Candidatus_Hodgkinia_cicadicola_Dsem_uid59311  Candidatus_Kinetoplastibacterium_blastocrithidii_TCC012E_uid189752  Candidatus_Kinetoplastibacterium_blastocrithidii__ex_Strigomonas_culicis__uid183629  Candidatus_Kinetoplastibacterium_crithidii_TCC036E_uid189749  Candidatus_Kinetoplastibacterium_crithidii__ex_Angomonas_deanei_ATCC_30255__uid183630  Candidatus_Kinetoplastibacterium_desouzaii_TCC079E_uid189750  Candidatus_Kinetoplastibacterium_galatii_TCC219_uid189751  Candidatus_Kinetoplastibacterium_oncopeltii_TCC290E_uid189840  Candidatus_Korarchaeum_cryptofilum_OPF8_uid58601  Candidatus_Koribacter_versatilis_Ellin345_uid58479  Candidatus_Liberibacter_americanus_Sao_Paulo_uid227424  Candidatus_Liberibacter_asiaticus_gxpsy_uid193764  Candidatus_Liberibacter_asiaticus_psy62_uid59227  Candidatus_Liberibacter_solanacearum_CLso_ZC1_uid61245  Candidatus_Methylomirabilis_oxyfera_uid161981  Candidatus_Midichloria_mitochondrii_IricVA_uid68687  Candidatus_Moranella_endobia_PCIT_uid68739  Candidatus_Moranella_endobia_PCVAL_uid197215  Candidatus_Mycoplasma_haemolamae_Purdue_uid171259  Candidatus_Mycoplasma_haemominutum__Birmingham_1__uid197195  Candidatus_Nasuia_deltocephalinicola_NAS_ALF_uid214084  Candidatus_Nitrosopumilus_AR2_uid176130  Candidatus_Nitrosopumilus_koreensis_AR1_uid176129  Candidatus_Nitrososphaera_gargensis_Ga9_2_uid176707  Candidatus_Nitrospira_defluvii_uid51175  Candidatus_Pelagibacter_IMCC9063_uid66305  Candidatus_Pelagibacter_ubique_HTCC1062_uid58401  Candidatus_Phytoplasma_australiense_uid61641  Candidatus_Phytoplasma_mali_uid59087  Candidatus_Phytoplasma_solani_284_09_uid225030  Candidatus_Portiera_aleyrodidarum_BT_B_uid173859  Candidatus_Portiera_aleyrodidarum_BT_B_uid176373  Candidatus_Portiera_aleyrodidarum_BT_QVLC_uid175570  Candidatus_Portiera_aleyrodidarum_BT_QVLC_uid176374  Candidatus_Portiera_aleyrodidarum_TV_uid195460  Candidatus_Protochlamydia_amoebophila_UWE25_uid58079  Candidatus_Puniceispirillum_marinum_IMCC1322_uid47081  Candidatus_Rickettsia_amblyommii_GAT_30V_uid156845  Candidatus_Riesia_pediculicola_USDA_uid46841  Candidatus_Ruthia_magnifica_Cm__Calyptogena_magnifica__uid58645  Candidatus_Saccharibacteria_bacterium_RAAC3_TM7_1_uid230715  Candidatus_Saccharobacterium_alaburgensis_uid203361  Candidatus_Solibacter_usitatus_Ellin6076_uid58139  Candidatus_Sulcia_muelleri_CARI_uid52535  Candidatus_Sulcia_muelleri_DMIN_uid47075  Candidatus_Sulcia_muelleri_GWSS_uid58943  Candidatus_Sulcia_muelleri_SMDSEM_uid59393  Candidatus_Sulcia_muelleri_Sulcia_ALF_uid214083  Candidatus_Tremblaya_phenacola_PAVE_uid209173  Candidatus_Tremblaya_princeps_PCIT_uid68741  Candidatus_Tremblaya_princeps_PCVAL_uid159519  Candidatus_Uzinura_diaspidicola_ASNER_uid186740  Candidatus_Vesicomyosocius_okutanii_HA_uid59427  Candidatus_Zinderia_insecticola_CARI_uid52459  Capnocytophaga_canimorsus_Cc5_uid70727  Capnocytophaga_ochracea_DSM_7271_uid59197  Carboxydothermus_hydrogenoformans_Z_2901_uid57821  Cardinium_endosymbiont_cEper1_of_Encarsia_pergandiella_uid175524  Carnobacterium_17_4_uid65789  Carnobacterium_WN1359_uid225603  Carnobacterium_maltaromaticum_LMA28_uid179370  Catenulispora_acidiphila_DSM_44928_uid59077  Caulobacter_K31_uid58551  Caulobacter_crescentus_CB15_uid57891  Caulobacter_crescentus_NA1000_uid59307  Caulobacter_segnis_ATCC_21756_uid41709  Cellulomonas_fimi_ATCC_484_uid66779  Cellulomonas_flavigena_DSM_20109_uid48821  Cellulophaga_algicola_DSM_14237_uid62159  Cellulophaga_lytica_DSM_7489_uid63401  Cellvibrio_japonicus_Ueda107_uid59139  Cenarchaeum_symbiosum_A_uid61411  Chamaesiphon_minutus_PCC_6605_uid183005  Chelativorans_BNC1_uid58069  Chitinophaga_pinensis_DSM_2588_uid59113  Chlamydia_muridarum_Nigg_uid57785  Chlamydia_pecorum_P787_uid221292  Chlamydia_pecorum_PV3056_3_uid221290  Chlamydia_pecorum_W73_uid221291  Chlamydia_psittaci_01DC12_uid179070  Chlamydia_psittaci_84_55_uid175571  Chlamydia_psittaci_CP3_uid175578  Chlamydia_psittaci_GR9_uid175572  Chlamydia_psittaci_M56_uid175576  Chlamydia_psittaci_MN_uid175573  Chlamydia_psittaci_NJ1_uid175579  Chlamydia_psittaci_VS225_uid175574  Chlamydia_psittaci_WC_uid175577  Chlamydia_psittaci_WS_RT_E30_uid175575  Chlamydia_trachomatis_434_Bu_uid61633  Chlamydia_trachomatis_A2497_uid159863  Chlamydia_trachomatis_A2497_uid159993  Chlamydia_trachomatis_A_363_uid196769  Chlamydia_trachomatis_A_5291_uid196770  Chlamydia_trachomatis_A_7249_uid196771  Chlamydia_trachomatis_A_HAR_13_uid58333  Chlamydia_trachomatis_B_Jali20_OT_uid59351  Chlamydia_trachomatis_B_TZ1A828_OT_uid59349  Chlamydia_trachomatis_C_TW_3_uid232251  Chlamydia_trachomatis_D_EC_uid159881  Chlamydia_trachomatis_D_LC_uid159879  Chlamydia_trachomatis_D_SotonD1_uid196772  Chlamydia_trachomatis_D_SotonD5_uid196773  Chlamydia_trachomatis_D_SotonD6_uid196774  Chlamydia_trachomatis_D_UW_3_CX_uid57637  Chlamydia_trachomatis_E_11023_uid161369  Chlamydia_trachomatis_E_150_uid161403  Chlamydia_trachomatis_E_Bour_uid196775  Chlamydia_trachomatis_E_C599_uid222812  Chlamydia_trachomatis_E_SW3_uid167483  Chlamydia_trachomatis_E_SotonE4_uid196776  Chlamydia_trachomatis_E_SotonE8_uid196777  Chlamydia_trachomatis_F_SW4_uid167484  Chlamydia_trachomatis_F_SW5_uid167485  Chlamydia_trachomatis_F_SWFPminus_uid222813  Chlamydia_trachomatis_G_11074_uid161409  Chlamydia_trachomatis_G_11222_uid161361  Chlamydia_trachomatis_G_9301_uid161377  Chlamydia_trachomatis_G_9768_uid161353  Chlamydia_trachomatis_G_SotonG1_uid196779  Chlamydia_trachomatis_IU824_uid193712  Chlamydia_trachomatis_IU888_uid193713  Chlamydia_trachomatis_Ia_SotonIa1_uid196780  Chlamydia_trachomatis_J_6276tet1_uid213394  Chlamydia_trachomatis_K_SotonK1_uid196782  Chlamydia_trachomatis_L1_115_uid196784  Chlamydia_trachomatis_L1_224_uid196785  Chlamydia_trachomatis_L1_440_LN_uid196783  Chlamydia_trachomatis_L2_25667R_uid196786  Chlamydia_trachomatis_L2_434_Bu_f__uid198644  Chlamydia_trachomatis_L2_434_Bu_i__uid198643  Chlamydia_trachomatis_L2b_795_uid196791  Chlamydia_trachomatis_L2b_8200_07_uid196787  Chlamydia_trachomatis_L2b_Ams1_uid196792  Chlamydia_trachomatis_L2b_Ams2_uid196793  Chlamydia_trachomatis_L2b_Ams3_uid196794  Chlamydia_trachomatis_L2b_Ams4_uid196795  Chlamydia_trachomatis_L2b_Ams5_uid196796  Chlamydia_trachomatis_L2b_CV204_uid196790  Chlamydia_trachomatis_L2b_Canada1_uid196798  Chlamydia_trachomatis_L2b_Canada2_uid196799  Chlamydia_trachomatis_L2b_LST_uid196789  Chlamydia_trachomatis_L2b_UCH_1_proctitis_uid61635  Chlamydia_trachomatis_L2b_UCH_2_uid196788  Chlamydia_trachomatis_L2c_uid68843  Chlamydia_trachomatis_L3_404_LN_uid196797  Chlamydia_trachomatis_RC_F_69_uid213385  Chlamydia_trachomatis_RC_F_s_342_uid213391  Chlamydia_trachomatis_RC_F_s_852_uid213387  Chlamydia_trachomatis_RC_J_943_uid213388  Chlamydia_trachomatis_RC_J_953_uid213389  Chlamydia_trachomatis_RC_J_966_uid213393  Chlamydia_trachomatis_RC_J_971_uid213395  Chlamydia_trachomatis_RC_J_s_122_uid213392  Chlamydia_trachomatis_RC_L2_55_uid213396  Chlamydia_trachomatis_RC_L2_s_3_uid213390  Chlamydia_trachomatis_RC_L2_s_46_uid213386  Chlamydia_trachomatis_Sweden2_uid161995  Chlamydia_trachomatis_uid196778  Chlamydia_trachomatis_uid196781  Chlamydia_trachomatis_uid196800  Chlamydia_trachomatis_uid216090  Chlamydophila_abortus_S26_3_uid57963  Chlamydophila_caviae_GPIC_uid57783  Chlamydophila_felis_Fe_C_56_uid57971  Chlamydophila_pecorum_E58_uid66295  Chlamydophila_pneumoniae_AR39_uid57809  Chlamydophila_pneumoniae_CWL029_uid57811  Chlamydophila_pneumoniae_J138_uid57829  Chlamydophila_pneumoniae_LPCoLN_uid159529  Chlamydophila_pneumoniae_TW_183_uid57997  Chlamydophila_psittaci_01DC11_uid159527  Chlamydophila_psittaci_02DC15_uid159521  Chlamydophila_psittaci_08DC60_uid159525  Chlamydophila_psittaci_6BC_uid159845  Chlamydophila_psittaci_6BC_uid63621  Chlamydophila_psittaci_C19_98_uid159523  Chlamydophila_psittaci_Mat116_uid189026  Chlamydophila_psittaci_RD1_uid162063  Chlorobaculum_parvum_NCIB_8327_uid59185  Chlorobium_chlorochromatii_CaD3_uid58375  Chlorobium_limicola_DSM_245_uid58127  Chlorobium_luteolum_DSM_273_uid58175  Chlorobium_phaeobacteroides_BS1_uid58131  Chlorobium_phaeobacteroides_DSM_266_uid58133  Chlorobium_phaeovibrioides_DSM_265_uid58129  Chlorobium_tepidum_TLS_uid57897  Chloroflexus_Y_400_fl_uid59085  Chloroflexus_aggregans_DSM_9485_uid58621  Chloroflexus_aurantiacus_J_10_fl_uid57657  Chloroherpeton_thalassium_ATCC_35110_uid59187  Chromobacterium_violaceum_ATCC_12472_uid58001  Chromohalobacter_salexigens_DSM_3043_uid62921  Chroococcidiopsis_thermalis_PCC_7203_uid183002  Chthonomonas_calidirosea_T49_uid208678  Citrobacter_koseri_ATCC_BAA_895_uid58143  Citrobacter_rodentium_ICC168_uid43089  Clavibacter_michiganensis_NCPPB_382_uid61625  Clavibacter_michiganensis_nebraskensis_NCPPB_2581_uid195908  Clavibacter_michiganensis_sepedonicus_uid61577  Clostridiales_genomosp__BVAB3_UPII9_5_uid46219  Clostridium_BNL1100_uid84307  Clostridium_SY8519_uid68705  Clostridium_acetobutylicum_ATCC_824_uid57677  Clostridium_acetobutylicum_DSM_1731_uid68293  Clostridium_acetobutylicum_EA_2018_uid159515  Clostridium_acidurici_9a_uid176126  Clostridium_autoethanogenum_DSM_10061_uid225029  Clostridium_beijerinckii_NCIMB_8052_uid58137  Clostridium_botulinum_A2_Kyoto_uid59229  Clostridium_botulinum_A3_Loch_Maree_uid59149  Clostridium_botulinum_A_ATCC_19397_uid58927  Clostridium_botulinum_A_ATCC_3502_uid61579  Clostridium_botulinum_A_Hall_uid58931  Clostridium_botulinum_B1_Okra_uid59147  Clostridium_botulinum_BKT015925_uid66203  Clostridium_botulinum_B_Eklund_17B_uid59159  Clostridium_botulinum_Ba4_657_uid59173  Clostridium_botulinum_E3_Alaska_E43_uid59157  Clostridium_botulinum_F_230613_uid159513  Clostridium_botulinum_F_Langeland_uid58929  Clostridium_botulinum_H04402_065_uid162091  Clostridium_cellulolyticum_H10_uid58709  Clostridium_cellulovorans_743B_uid51503  Clostridium_cf__saccharolyticum_K10_uid197201  Clostridium_clariflavum_DSM_19732_uid82345  Clostridium_difficile_630_uid57679  Clostridium_difficile_BI1_uid158363  Clostridium_difficile_CD196_uid41017  Clostridium_difficile_R20291_uid40921  Clostridium_kluyveri_DSM_555_uid58885  Clostridium_kluyveri_NBRC_12016_uid59369  Clostridium_lentocellum_DSM_5427_uid49117  Clostridium_ljungdahlii_DSM_13528_uid50583  Clostridium_novyi_NT_uid58643  Clostridium_pasteurianum_BC1_uid201478  Clostridium_perfringens_13_uid57681  Clostridium_perfringens_ATCC_13124_uid57901  Clostridium_perfringens_SM101_uid58117  Clostridium_phytofermentans_ISDg_uid58519  Clostridium_saccharobutylicum_DSM_13864_uid223284  Clostridium_saccharolyticum_WM1_uid51419  Clostridium_saccharoperbutylacetonicum_ATCC_27021_uid189747  Clostridium_stercorarium_DSM_8532_uid186819  Clostridium_stercorarium_DSM_8532_uid195569  Clostridium_tetani_12124569_uid227214  Clostridium_tetani_E88_uid57683  Clostridium_thermocellum_ATCC_27405_uid57917  Clostridium_thermocellum_DSM_1313_uid161989  Collimonas_fungivorans_Ter331_uid70793  Colwellia_psychrerythraea_34H_uid57855  Comamonadaceae_bacterium_CR_uid223378  Comamonas_testosteroni_CNB_2_uid62961  Conexibacter_woesei_DSM_14684_uid43467  Coprococcus_ART55_1_uid197176  Coprococcus_catus_GD_7_uid197174  Coprothermobacter_proteolyticus_DSM_5265_uid59253  Coraliomargarita_akajimensis_DSM_45221_uid47079  Corallococcus_coralloides_DSM_2259_uid157997  Coriobacterium_glomerans_PW2_uid65787  Corynebacterium_argentoratense_DSM_44202_uid217419  Corynebacterium_aurimucosum_ATCC_700975_uid59409  Corynebacterium_callunae_DSM_20147_uid193714  Corynebacterium_diphtheriae_241_uid83607  Corynebacterium_diphtheriae_31A_uid84309  Corynebacterium_diphtheriae_BH8_uid84311  Corynebacterium_diphtheriae_C7__beta__uid84313  Corynebacterium_diphtheriae_CDCE_8392_uid84295  Corynebacterium_diphtheriae_HC01_uid84297  Corynebacterium_diphtheriae_HC02_uid84317  Corynebacterium_diphtheriae_HC03_uid84299  Corynebacterium_diphtheriae_HC04_uid84301  Corynebacterium_diphtheriae_INCA_402_uid83605  Corynebacterium_diphtheriae_NCTC_13129_uid57691  Corynebacterium_diphtheriae_PW8_uid84303  Corynebacterium_diphtheriae_VA01_uid84305  Corynebacterium_efficiens_YS_314_uid62905  Corynebacterium_glutamicum_ATCC_13032_uid193708  Corynebacterium_glutamicum_ATCC_13032_uid57905  Corynebacterium_glutamicum_ATCC_13032_uid61611  Corynebacterium_glutamicum_MB001_uid214793  Corynebacterium_glutamicum_R_uid58897  Corynebacterium_glutamicum_SCgG1_uid207285  Corynebacterium_glutamicum_SCgG2_uid207286  Corynebacterium_halotolerans_YIM_70093___DSM_44683_uid189953  Corynebacterium_jeikeium_K411_uid58399  Corynebacterium_kroppenstedtii_DSM_44385_uid59411  Corynebacterium_maris_DSM_45190_uid214081  Corynebacterium_pseudotuberculosis_1002_uid159677  Corynebacterium_pseudotuberculosis_1_06_A_uid159665  Corynebacterium_pseudotuberculosis_258_uid167260  Corynebacterium_pseudotuberculosis_267_uid162175  Corynebacterium_pseudotuberculosis_316_uid89381  Corynebacterium_pseudotuberculosis_31_uid162167  Corynebacterium_pseudotuberculosis_3_99_5_uid83609  Corynebacterium_pseudotuberculosis_42_02_A_uid159669  Corynebacterium_pseudotuberculosis_C231_uid159675  Corynebacterium_pseudotuberculosis_CIP_52_97_uid159667  Corynebacterium_pseudotuberculosis_Cp162_uid168258  Corynebacterium_pseudotuberculosis_FRC41_uid50585  Corynebacterium_pseudotuberculosis_I19_uid159673  Corynebacterium_pseudotuberculosis_P54B96_uid157909  Corynebacterium_pseudotuberculosis_PAT10_uid159671  Corynebacterium_resistens_DSM_45100_uid50555  Corynebacterium_terpenotabidum_Y_11_uid210639  Corynebacterium_ulcerans_0102_uid169879  Corynebacterium_ulcerans_809_uid159659  Corynebacterium_ulcerans_BR_AD22_uid68291  Corynebacterium_urealyticum_DSM_7109_uid61639  Corynebacterium_urealyticum_DSM_7111_uid188688  Corynebacterium_variabile_DSM_44702_uid62003  Coxiella_burnetii_CbuG_Q212_uid58893  Coxiella_burnetii_CbuK_Q154_uid58895  Coxiella_burnetii_Dugway_5J108_111_uid58629  Coxiella_burnetii_RSA_331_uid58637  Coxiella_burnetii_RSA_493_uid57631  Crinalium_epipsammum_PCC_9333_uid183113  Croceibacter_atlanticus_HTCC2559_uid49661  Cronobacter_sakazakii_45402_uid231516  Cronobacter_sakazakii_ATCC_BAA_894_uid58145  Cronobacter_sakazakii_ES15_uid167045  Cronobacter_sakazakii_Sp291_uid189241  Cronobacter_turicensis_z3032_uid40821  Cryptobacterium_curtum_DSM_15641_uid59041  Cupriavidus_metallidurans_CH34_uid57815  Cupriavidus_necator_N_1_uid68689  Cupriavidus_taiwanensis_LMG_19424_uid61615  Cyanobacterium_aponinum_PCC_10605_uid183340  Cyanobacterium_stanieri_PCC_7202_uid183337  Cyanobium_gracile_PCC_6307_uid182931  Cyanothece_ATCC_51142_uid59013  Cyanothece_PCC_7424_uid59025  Cyanothece_PCC_7425_uid59435  Cyanothece_PCC_7822_uid52547  Cyanothece_PCC_8801_uid59027  Cyanothece_PCC_8802_uid59143  Cyclobacterium_marinum_DSM_745_uid71485  Cycloclasticus_P1_uid176368  Cycloclasticus_zancles_7_ME_uid214092  Cylindrospermum_stagnale_PCC_7417_uid183111  Cytophaga_hutchinsonii_ATCC_33406_uid57651  Dactylococcopsis_salina_PCC_8305_uid183341  Dechloromonas_aromatica_RCB_uid58025  Dechlorosoma_suillum_PS_uid81439  Deferribacter_desulfuricans_SSM1_uid46653  Dehalobacter_11DCA_uid177715  Dehalobacter_CF_uid177714  Dehalococcoides_BAV1_uid58477  Dehalococcoides_CBDB1_uid58413  Dehalococcoides_GT_uid42115  Dehalococcoides_VS_uid42393  Dehalococcoides_ethenogenes_195_uid57763  Dehalococcoides_mccartyi_BTF08_uid190183  Dehalococcoides_mccartyi_DCMB5_uid190184  Dehalococcoides_mccartyi_GY50_uid230266  Dehalogenimonas_lykanthroporepellens_BL_DC_9_uid48131  Deinococcus_deserti_VCD115_uid58615  Deinococcus_geothermalis_DSM_11300_uid58275  Deinococcus_gobiensis_I_0_uid162509  Deinococcus_maricopensis_DSM_21211_uid62225  Deinococcus_peraridilitoris_DSM_19664_uid183485  Deinococcus_proteolyticus_MRP_uid63399  Deinococcus_radiodurans_R1_uid57665  Delftia_Cs1_4_uid67319  Delftia_acidovorans_SPH_1_uid58703  Denitrovibrio_acetiphilus_DSM_12809_uid46657  Desulfarculus_baarsii_DSM_2075_uid51371  Desulfatibacillum_alkenivorans_AK_01_uid58913  Desulfitobacterium_dehalogenans_ATCC_51507_uid82553  Desulfitobacterium_dichloroeliminans_LMG_P_21439_uid82555  Desulfitobacterium_hafniense_DCB_2_uid57749  Desulfitobacterium_hafniense_Y51_uid58605  Desulfobacca_acetoxidans_DSM_11109_uid65785  Desulfobacterium_autotrophicum_HRM2_uid59061  Desulfobacula_toluolica_Tol2_uid175777  Desulfobulbus_propionicus_DSM_2032_uid62265  Desulfocapsa_sulfexigens_DSM_10523_uid189952  Desulfococcus_oleovorans_Hxd3_uid58777  Desulfohalobium_retbaense_DSM_5692_uid59183  Desulfomicrobium_baculatum_DSM_4028_uid59217  Desulfomonile_tiedjei_DSM_6799_uid168320  Desulfosporosinus_acidiphilus_SJ4_uid156759  Desulfosporosinus_meridiei_DSM_13257_uid75097  Desulfosporosinus_orientis_DSM_765_uid82939  Desulfotalea_psychrophila_LSv54_uid58153  Desulfotomaculum_acetoxidans_DSM_771_uid59109  Desulfotomaculum_carboxydivorans_CO_1_SRB_uid67317  Desulfotomaculum_gibsoniae_DSM_7213_uid76945  Desulfotomaculum_kuznetsovii_DSM_6115_uid67357  Desulfotomaculum_reducens_MI_1_uid58277  Desulfotomaculum_ruminis_DSM_2154_uid67507  Desulfovibrio_aespoeensis_Aspo_2_uid42613  Desulfovibrio_africanus_Walvis_Bay_uid66847  Desulfovibrio_alaskensis_G20_uid57941  Desulfovibrio_desulfuricans_ATCC_27774_uid59213  Desulfovibrio_desulfuricans_ND132_uid63159  Desulfovibrio_gigas_DSM_1382_uid221293  Desulfovibrio_hydrothermalis_AM13___DSM_14728_uid184831  Desulfovibrio_magneticus_RS_1_uid59309  Desulfovibrio_piezophilus_C1TLV30_uid190704  Desulfovibrio_salexigens_DSM_2638_uid59223  Desulfovibrio_vulgaris_DP4_uid58679  Desulfovibrio_vulgaris_Hildenborough_uid57645  Desulfovibrio_vulgaris_RCH1_uid161961  Desulfovibrio_vulgaris__Miyazaki_F__uid59089  Desulfurispirillum_indicum_S5_uid45897  Desulfurivibrio_alkaliphilus_AHT2_uid49487  Desulfurobacterium_thermolithotrophum_DSM_11699_uid63405  Desulfurococcus_fermentans_DSM_16532_uid75119  Desulfurococcus_kamchatkensis_1221n_uid59133  Desulfurococcus_mucosus_DSM_2162_uid62227  Dichelobacter_nodosus_VCS1703A_uid57643  Dickeya_dadantii_3937_uid52537  Dickeya_dadantii_Ech586_uid42519  Dickeya_dadantii_Ech703_uid59363  Dickeya_zeae_Ech1591_uid59297  Dictyoglomus_thermophilum_H_6_12_uid59439  Dictyoglomus_turgidum_DSM_6724_uid59177  Dinoroseobacter_shibae_DFL_12_uid58707  Dyadobacter_fermentans_DSM_18053_uid59049  Echinicola_vietnamensis_DSM_17526_uid184076  Ectothiorhodospiraceae_bacterium_M19_40_uid199898  Edwardsiella_ictaluri_93_146_uid59403  Edwardsiella_tarda_C07_087_uid193773  Edwardsiella_tarda_EIB202_uid41819  Edwardsiella_tarda_FL6_60_uid159657  Eggerthella_YY7918_uid68707  Eggerthella_lenta_DSM_2243_uid59079  Ehrlichia_canis_Jake_uid58071  Ehrlichia_chaffeensis_Arkansas_uid57933  Ehrlichia_muris_AS145_uid232250  Ehrlichia_ruminantium_Gardel_uid58245  Ehrlichia_ruminantium_Welgevonden_uid58013  Ehrlichia_ruminantium_Welgevonden_uid58243  Elusimicrobium_minutum_Pei191_uid58949  Emticicia_oligotrophica_DSM_17448_uid177079  Enterobacter_638_uid58727  Enterobacter_R4_368_uid208672  Enterobacter_aerogenes_EA1509E_uid187411  Enterobacter_aerogenes_KCTC_2190_uid68103  Enterobacter_asburiae_LF7a_uid72793  Enterobacter_cloacae_ATCC_13047_uid48363  Enterobacter_cloacae_ENHKU01_uid172463  Enterobacter_cloacae_EcWSU1_uid80739  Enterobacter_cloacae_NCTC_9394_uid197202  Enterobacter_cloacae_SCF1_uid59969  Enterobacter_cloacae_dissolvens_SDM_uid168997  Enterobacteriaceae_bacterium_FGI_57_uid185181  Enterococcus_7L76_uid197170  Enterococcus_casseliflavus_EC20_uid55693  Enterococcus_faecalis_62_uid159663  Enterococcus_faecalis_D32_uid171261  Enterococcus_faecalis_OG1RF_uid54927  Enterococcus_faecalis_Symbioflor_1_uid183342  Enterococcus_faecalis_V583_uid57669  Enterococcus_faecium_Aus0004_uid87025  Enterococcus_faecium_Aus0085_uid214432  Enterococcus_faecium_DO_uid55353  Enterococcus_faecium_NRRL_B_2354_uid188477  Enterococcus_hirae_ATCC_9790_uid70619  Enterococcus_mundtii_QU_25_uid229420  Erwinia_Ejp617_uid159955  Erwinia_amylovora_ATCC_49946_uid46943  Erwinia_amylovora_CFBP1430_uid46839  Erwinia_billingiae_Eb661_uid50547  Erwinia_pyrifoliae_DSM_12163_uid159693  Erwinia_pyrifoliae_Ep1_96_uid40659  Erwinia_tasmaniensis_Et1_99_uid59029  Erysipelothrix_rhusiopathiae_Fujisawa_uid68021  Erysipelothrix_rhusiopathiae_SY1027_uid206518  Erythrobacter_litoralis_HTCC2594_uid58299  Escherichia_blattae_DSM_4481_uid165043  Escherichia_coli_042_uid161985  Escherichia_coli_536_uid58531  Escherichia_coli_55989_uid59383  Escherichia_coli_ABU_83972_uid161975  Escherichia_coli_APEC_O1_uid58623  Escherichia_coli_APEC_O78_uid187277  Escherichia_coli_ATCC_8739_uid58783  Escherichia_coli_BL21_DE3__uid161947  Escherichia_coli_BL21_DE3__uid161949  Escherichia_coli_BW2952_uid59391  Escherichia_coli_B_REL606_uid58803  Escherichia_coli_CFT073_uid57915  Escherichia_coli_DH1_uid161951  Escherichia_coli_DH1_uid162051  Escherichia_coli_E24377A_uid58395  Escherichia_coli_ED1a_uid59379  Escherichia_coli_ETEC_H10407_uid161993  Escherichia_coli_HS_uid58393  Escherichia_coli_IAI1_uid59377  Escherichia_coli_IAI39_uid59381  Escherichia_coli_IHE3034_uid162007  Escherichia_coli_JJ1886_uid226103  Escherichia_coli_KO11FL_uid162099  Escherichia_coli_KO11FL_uid52593  Escherichia_coli_K_12_substr__DH10B_uid58979  Escherichia_coli_K_12_substr__MDS42_uid193705  Escherichia_coli_K_12_substr__MG1655_uid57779  Escherichia_coli_K_12_substr__W3110_uid161931  Escherichia_coli_LF82_uid161965  Escherichia_coli_LY180_uid219461  Escherichia_coli_NA114_uid162139  Escherichia_coli_O103_H2_12009_uid41013  Escherichia_coli_O104_H4_2009EL_2050_uid175905  Escherichia_coli_O104_H4_2009EL_2071_uid176128  Escherichia_coli_O104_H4_2011C_3493_uid176127  Escherichia_coli_O111_H__11128_uid41023  Escherichia_coli_O127_H6_E2348_69_uid59343  Escherichia_coli_O157_H7_EC4115_uid59091  Escherichia_coli_O157_H7_EDL933_uid57831  Escherichia_coli_O157_H7_TW14359_uid59235  Escherichia_coli_O157_H7_uid57781  Escherichia_coli_O26_H11_11368_uid41021  Escherichia_coli_O55_H7_CB9615_uid46655  Escherichia_coli_O55_H7_RM12579_uid162153  Escherichia_coli_O7_K1_CE10_uid162115  Escherichia_coli_O83_H1_NRG_857C_uid161987  Escherichia_coli_P12b_uid162061  Escherichia_coli_PMV_1_uid219679  Escherichia_coli_S88_uid62979  Escherichia_coli_SE11_uid59425  Escherichia_coli_SE15_uid161939  Escherichia_coli_SMS_3_5_uid58919  Escherichia_coli_UM146_uid162043  Escherichia_coli_UMN026_uid62981  Escherichia_coli_UMNK88_uid161991  Escherichia_coli_UTI89_uid58541  Escherichia_coli_W_uid162011  Escherichia_coli_W_uid162101  Escherichia_coli_Xuzhou21_uid163995  Escherichia_coli__BL21_Gold_DE3_pLysS_AG__uid59245  Escherichia_coli__clone_D_i14__uid162049  Escherichia_coli__clone_D_i2__uid162047  Escherichia_fergusonii_ATCC_35469_uid59375  Ethanoligenens_harbinense_YUAN_3_uid46255  Eubacterium_cylindroides_T2_87_uid197177  Eubacterium_eligens_ATCC_27750_uid59171  Eubacterium_limosum_KIST612_uid59777  Eubacterium_rectale_ATCC_33656_uid59169  Eubacterium_rectale_uid197161  Eubacterium_rectale_uid197162  Eubacterium_siraeum_V10Sc8a_uid197178  Eubacterium_siraeum_uid197160  Exiguobacterium_AT1b_uid59093  Exiguobacterium_MH3_uid227425  Exiguobacterium_antarcticum_B7_uid176125  Exiguobacterium_sibiricum_255_15_uid58053  Faecalibacterium_prausnitzii_L2_6_uid197183  Faecalibacterium_prausnitzii_uid197157  Ferrimonas_balearica_DSM_9799_uid53371  Ferroglobus_placidus_DSM_10642_uid40863  Ferroplasma_acidarmanus_fer1_uid54095  Fervidicoccus_fontis_Kam940_uid162201  Fervidobacterium_nodosum_Rt17_B1_uid58625  Fervidobacterium_pennivorans_DSM_9078_uid78143  Fibrella_aestuarina_uid178352  Fibrobacter_succinogenes_S85_uid161919  Fibrobacter_succinogenes_S85_uid41169  Filifactor_alocis_ATCC_35896_uid46625  Finegoldia_magna_ATCC_29328_uid58867  Flavobacteriaceae_bacterium_3519_10_uid59413  Flavobacteriales_bacterium_HTCC2170_uid51877  Flavobacterium_branchiophilum_FL_15_uid73421  Flavobacterium_columnare_ATCC_49512_uid80731  Flavobacterium_indicum_GPTSA100_9_uid157999  Flavobacterium_johnsoniae_UW101_uid58493  Flavobacterium_psychrophilum_JIP02_86_uid61627  Flexibacter_litoralis_DSM_6794_uid168257  Flexistipes_sinusarabici_DSM_4947_uid68147  Fluviicola_taffensis_DSM_16823_uid65271  Francisella_TX077308_uid68321  Francisella_cf__novicida_3523_uid162107  Francisella_cf__novicida_Fx1_uid162105  Francisella_noatunensis_orientalis_LADL_07_285A_uid231515  Francisella_noatunensis_orientalis_Toba_04_uid164779  Francisella_novicida_U112_uid58499  Francisella_philomiragia_ATCC_25017_uid59105  Francisella_tularensis_FSC198_uid58693  Francisella_tularensis_NE061598_uid161973  Francisella_tularensis_SCHU_S4_uid57589  Francisella_tularensis_TI0902_uid89373  Francisella_tularensis_TIGB03_uid89379  Francisella_tularensis_WY96_3418_uid58811  Francisella_tularensis_holarctica_F92_uid181998  Francisella_tularensis_holarctica_FSC200_uid54341  Francisella_tularensis_holarctica_FTNF002_00_uid58999  Francisella_tularensis_holarctica_LVS_uid58595  Francisella_tularensis_holarctica_OSU18_uid58687  Francisella_tularensis_mediasiatica_FSC147_uid58939  Frankia_CcI3_uid58397  Frankia_EAN1pec_uid58367  Frankia_EuI1c_uid42615  Frankia_alni_ACN14a_uid58695  Frankia_symbiont_of_Datisca_glomerata_uid46257  Frateuria_aurantia_DSM_6220_uid81775  Fusobacterium_3_1_36A2_uid55995  Fusobacterium_4_8_uid205051  Fusobacterium_nucleatum_ATCC_25586_uid57885  Fusobacterium_nucleatum_polymorphum_ATCC_10953_uid54419  Gallibacterium_anatis_UMN179_uid66567  Gallionella_capsiferriformans_ES_2_uid51505  Gardnerella_vaginalis_409_05_uid43211  Gardnerella_vaginalis_ATCC_14019_uid55487  Gardnerella_vaginalis_HMP9231_uid162045  Geitlerinema_PCC_7407_uid183007  Gemmatimonas_aurantiaca_T_27_uid58813  Geobacillus_C56_T3_uid49467  Geobacillus_HH01_uid188479  Geobacillus_JF8_uid215234  Geobacillus_WCH70_uid59045  Geobacillus_Y412MC52_uid55381  Geobacillus_Y412MC61_uid41171  Geobacillus_Y4_1MC1_uid55779  Geobacillus_kaustophilus_HTA426_uid58227  Geobacillus_thermodenitrificans_NG80_2_uid58829  Geobacillus_thermoglucosidasius_C56_YS93_uid48129  Geobacillus_thermoleovorans_CCB_US3_UF5_uid82949  Geobacter_FRC_32_uid58543  Geobacter_M18_uid55771  Geobacter_M21_uid59037  Geobacter_bemidjiensis_Bem_uid58749  Geobacter_lovleyi_SZ_uid58713  Geobacter_metallireducens_GS_15_uid57731  Geobacter_sulfurreducens_KN400_uid161977  Geobacter_sulfurreducens_PCA_uid57743  Geobacter_uraniireducens_Rf4_uid58475  Geodermatophilus_obscurus_DSM_43160_uid43725  Glaciecola_4H_3_7_YE_5_uid66595  Glaciecola_nitratireducens_FR1064_uid73759  Glaciecola_psychrophila_170_uid193711  Gloeobacter_JS_uid225602  Gloeobacter_violaceus_PCC_7421_uid58011  Gloeocapsa_PCC_7428_uid183112  Gluconacetobacter_diazotrophicus_PAl_5_uid59075  Gluconacetobacter_diazotrophicus_PAl_5_uid61587  Gluconacetobacter_xylinus_NBRC_3288_uid46523  Gluconobacter_oxydans_621H_uid58239  Gluconobacter_oxydans_H24_uid179202  Gordonia_KTR9_uid174812  Gordonia_bronchialis_DSM_43247_uid41403  Gordonia_polyisoprenivorans_VH2_uid86651  Gordonibacter_pamelaeae_7_10_1_b_uid197167  Gramella_forsetii_KT0803_uid58881  Granulibacter_bethesdensis_CGDNIH1_uid58661  Granulicella_mallensis_MP5ACTX8_uid49957  Haemophilus_ducreyi_35000HP_uid57625  Haemophilus_influenzae_10810_uid86647  Haemophilus_influenzae_86_028NP_uid58093  Haemophilus_influenzae_F3031_uid62123  Haemophilus_influenzae_F3047_uid62097  Haemophilus_influenzae_KR494_uid219323  Haemophilus_influenzae_PittEE_uid58591  Haemophilus_influenzae_PittGG_uid58593  Haemophilus_influenzae_R2846_uid161921  Haemophilus_influenzae_R2866_uid161923  Haemophilus_influenzae_Rd_KW20_uid57771  Haemophilus_parainfluenzae_T3T1_uid72801  Haemophilus_parasuis_SH0165_uid59273  Haemophilus_parasuis_ZJ0906_uid209117  Haemophilus_somnus_129PT_uid57929  Haemophilus_somnus_2336_uid57979  Hahella_chejuensis_KCTC_2396_uid58483  Halalkalicoccus_jeotgali_B3_uid50305  Halanaerobium_hydrogeniformans_uid60191  Halanaerobium_praevalens_DSM_2228_uid161959  Haliangium_ochraceum_DSM_14365_uid41425  Haliscomenobacter_hydrossis_DSM_1100_uid66777  Haloarcula_hispanica_ATCC_33960_uid72475  Haloarcula_hispanica_N601_uid230920  Haloarcula_marismortui_ATCC_43049_uid57719  Halobacillus_halophilus_DSM_2266_uid162033  Halobacterium_NRC_1_uid57769  Halobacterium_salinarum_R1_uid61571  Halobacteroides_halobius_DSM_5150_uid184862  Haloferax_mediterranei_ATCC_33500_uid167315  Haloferax_volcanii_DS2_uid46845  Halogeometricum_borinquense_DSM_11551_uid54919  Halomicrobium_mukohataei_DSM_12286_uid59107  Halomonas_elongata_DSM_2581_uid52781  Halopiger_xanaduensis_SH_6_uid68105  Haloquadratum_walsbyi_C23_uid162019  Haloquadratum_walsbyi_DSM_16790_uid58673  Halorhabdus_tiamatea_SARL4B_uid214082  Halorhabdus_utahensis_DSM_12940_uid59189  Halorhodospira_halophila_SL1_uid58473  Halorubrum_lacusprofundi_ATCC_49239_uid58807  Haloterrigena_turkmenica_DSM_5511_uid43501  Halothece_PCC_7418_uid183338  Halothermothrix_orenii_H_168_uid58585  Halothiobacillus_neapolitanus_c2_uid41317  Halovivax_ruber_XH_70_uid184819  Halyomorpha_halys_symbiont_uid222821  Helicobacter_acinonychis_Sheeba_uid58685  Helicobacter_bizzozeronii_CIII_1_uid68141  Helicobacter_cetorum_MIT_00_7128_uid162217  Helicobacter_cetorum_MIT_99_5656_uid162215  Helicobacter_cinaedi_ATCC_BAA_847_uid193765  Helicobacter_cinaedi_PAGU611_uid162219  Helicobacter_felis_ATCC_49179_uid61409  Helicobacter_heilmannii_ASB1_4_uid182935  Helicobacter_hepaticus_ATCC_51449_uid57737  Helicobacter_mustelae_12198_uid46647  Helicobacter_pylori_2017_uid161151  Helicobacter_pylori_2018_uid161159  Helicobacter_pylori_26695_uid178201  Helicobacter_pylori_26695_uid57787  Helicobacter_pylori_35A_uid49903  Helicobacter_pylori_51_uid161925  Helicobacter_pylori_83_uid161153  Helicobacter_pylori_908_uid159985  Helicobacter_pylori_Aklavik117_uid182201  Helicobacter_pylori_Aklavik86_uid182202  Helicobacter_pylori_B38_uid59415  Helicobacter_pylori_B8_uid49873  Helicobacter_pylori_BM012A_uid229744  Helicobacter_pylori_BM012S_uid229881  Helicobacter_pylori_Cuz20_uid159987  Helicobacter_pylori_ELS37_uid158157  Helicobacter_pylori_F16_uid161145  Helicobacter_pylori_F30_uid159991  Helicobacter_pylori_F32_uid161139  Helicobacter_pylori_F57_uid161143  Helicobacter_pylori_G27_uid59305  Helicobacter_pylori_Gambia94_24_uid159493  Helicobacter_pylori_HPAG1_uid58517  Helicobacter_pylori_HUP_B14_uid162213  Helicobacter_pylori_India7_uid161149  Helicobacter_pylori_J99_uid57789  Helicobacter_pylori_Lithuania75_uid159491  Helicobacter_pylori_OK113_uid193715  Helicobacter_pylori_OK310_uid193716  Helicobacter_pylori_P12_uid59327  Helicobacter_pylori_PeCan18_uid162211  Helicobacter_pylori_PeCan4_uid53539  Helicobacter_pylori_Puno120_uid159611  Helicobacter_pylori_Puno135_uid161157  Helicobacter_pylori_Rif1_uid178202  Helicobacter_pylori_Rif2_uid178203  Helicobacter_pylori_SJM180_uid53541  Helicobacter_pylori_SNT49_uid159615  Helicobacter_pylori_Sat464_uid159467  Helicobacter_pylori_Shi112_uid162207  Helicobacter_pylori_Shi169_uid162209  Helicobacter_pylori_Shi417_uid162205  Helicobacter_pylori_Shi470_uid59165  Helicobacter_pylori_SouthAfrica20_uid216150  Helicobacter_pylori_SouthAfrica7_uid159989  Helicobacter_pylori_UM032_uid203025  Helicobacter_pylori_UM037_uid203027  Helicobacter_pylori_UM066_uid203028  Helicobacter_pylori_UM298_uid213226  Helicobacter_pylori_UM299_uid203026  Helicobacter_pylori_XZ274_uid165869  Helicobacter_pylori_uid159983  Helicobacter_pylori_v225d_uid159639  Heliobacterium_modesticaldum_Ice1_uid58279  Herbaspirillum_seropedicae_SmR1_uid50427  Herminiimonas_arsenicoxydans_uid58291  Herpetosiphon_aurantiacus_DSM_785_uid58599  Hippea_maritima_DSM_10411_uid65267  Hirschia_baltica_ATCC_49814_uid59365  Hydrogenobacter_thermophilus_TK_6_uid159875  Hydrogenobacter_thermophilus_TK_6_uid45927  Hydrogenobaculum_HO_uid190882  Hydrogenobaculum_SN_uid46251  Hydrogenobaculum_Y04AAS1_uid58857  Hyperthermus_butylicus_DSM_5456_uid57755  Hyphomicrobium_MC1_uid68453  Hyphomicrobium_denitrificans_1NES1_uid179904  Hyphomicrobium_denitrificans_ATCC_51888_uid50325  Hyphomicrobium_nitrativorans_NL23_uid230615  Hyphomonas_neptunium_ATCC_15444_uid58433  Idiomarina_loihiensis_GSL_199_uid205256  Idiomarina_loihiensis_L2TR_uid58087  Ignavibacterium_album_JCM_16511_uid162097  Ignicoccus_hospitalis_KIN4_I_uid58365  Ignisphaera_aggregans_DSM_17230_uid51875  Ilyobacter_polytropus_DSM_2926_uid59769  Intrasporangium_calvum_DSM_43043_uid61729  Isoptericola_variabilis_225_uid67501  Isosphaera_pallida_ATCC_43644_uid62207  Jannaschia_CCS1_uid58147  Janthinobacterium_Marseille_uid58603  Jonesia_denitrificans_DSM_20603_uid59053  Kangiella_koreensis_DSM_16069_uid59209  Ketogulonicigenium_vulgare_WSH_001_uid161161  Ketogulonicigenium_vulgare_Y25_uid59581  Kineococcus_radiotolerans_SRS30216_uid58067  Kitasatospora_setae_KM_6054_uid77027  Klebsiella_oxytoca_E718_uid170256  Klebsiella_oxytoca_KCTC_1686_uid83159  Klebsiella_pneumoniae_1084_uid174151  Klebsiella_pneumoniae_342_uid59145  Klebsiella_pneumoniae_CG43_uid223021  Klebsiella_pneumoniae_HS11286_uid84387  Klebsiella_pneumoniae_JM45_uid215235  Klebsiella_pneumoniae_KCTC_2242_uid162147  Klebsiella_pneumoniae_MGH_78578_uid57619  Klebsiella_pneumoniae_NTUH_K2044_uid59073  Klebsiella_pneumoniae_rhinoscleromatis_SB3432_uid203334  Klebsiella_variicola_At_22_uid42113  Kocuria_rhizophila_DC2201_uid59099  Kosmotoga_olearia_TBF_19_5_1_uid59205  Kribbella_flavida_DSM_17836_uid43465  Krokinobacter_4H_3_7_5_uid66593  Kyrpidia_tusciae_DSM_2912_uid48361  Kytococcus_sedentarius_DSM_20547_uid59071  Lacinutrix_5H_3_7_4_uid68067  Lactobacillus_acidophilus_30SC_uid63605  Lactobacillus_acidophilus_La_14_uid201479  Lactobacillus_acidophilus_NCFM_uid57685  Lactobacillus_amylovorus_GRL1118_uid160233  Lactobacillus_amylovorus_GRL_1112_uid61179  Lactobacillus_brevis_ATCC_367_uid57989  Lactobacillus_brevis_KB290_uid195560  Lactobacillus_buchneri_NRRL_B_30929_uid66205  Lactobacillus_buchneri_uid73657  Lactobacillus_casei_ATCC_334_uid57985  Lactobacillus_casei_BD_II_uid162119  Lactobacillus_casei_BL23_uid59237  Lactobacillus_casei_LC2W_uid162121  Lactobacillus_casei_LOCK919_uid210959  Lactobacillus_casei_W56_uid178736  Lactobacillus_casei_Zhang_uid50673  Lactobacillus_crispatus_ST1_uid48359  Lactobacillus_delbrueckii_bulgaricus_2038_uid161929  Lactobacillus_delbrueckii_bulgaricus_ATCC_11842_uid58647  Lactobacillus_delbrueckii_bulgaricus_ATCC_BAA_365_uid57987  Lactobacillus_delbrueckii_bulgaricus_ND02_uid60621  Lactobacillus_fermentum_CECT_5716_uid162003  Lactobacillus_fermentum_F6_uid203391  Lactobacillus_fermentum_IFO_3956_uid58865  Lactobacillus_gasseri_ATCC_33323_uid57687  Lactobacillus_helveticus_CNRZ32_uid212302  Lactobacillus_helveticus_DPC_4571_uid58761  Lactobacillus_helveticus_H10_uid162017  Lactobacillus_helveticus_R0052_uid174439  Lactobacillus_johnsonii_DPC_6026_uid162057  Lactobacillus_johnsonii_FI9785_uid41735  Lactobacillus_johnsonii_N6_2_uid229876  Lactobacillus_johnsonii_NCC_533_uid58029  Lactobacillus_kefiranofaciens_ZW3_uid67985  Lactobacillus_paracasei_8700_2_uid55295  Lactobacillus_plantarum_16_uid209042  Lactobacillus_plantarum_JDM1_uid59361  Lactobacillus_plantarum_P8_uid203333  Lactobacillus_plantarum_ST_III_uid53537  Lactobacillus_plantarum_WCFS1_uid62911  Lactobacillus_plantarum_ZJ316_uid188689  Lactobacillus_reuteri_DSM_20016_uid58471  Lactobacillus_reuteri_I5007_uid208677  Lactobacillus_reuteri_JCM_1112_uid58875  Lactobacillus_reuteri_SD2112_uid55357  Lactobacillus_reuteri_TD1_uid213089  Lactobacillus_rhamnosus_ATCC_8530_uid162169  Lactobacillus_rhamnosus_GG_uid161983  Lactobacillus_rhamnosus_GG_uid59313  Lactobacillus_rhamnosus_LOCK900_uid210957  Lactobacillus_rhamnosus_LOCK908_uid210958  Lactobacillus_rhamnosus_Lc_705_uid59315  Lactobacillus_ruminis_ATCC_27782_uid73417  Lactobacillus_sakei_23K_uid58281  Lactobacillus_salivarius_CECT_5713_uid162005  Lactobacillus_salivarius_UCC118_uid58233  Lactobacillus_sanfranciscensis_TMW_1_1304_uid72937  Lactococcus_garvieae_ATCC_49156_uid73413  Lactococcus_garvieae_Lg2_uid161935  Lactococcus_lactis_CV56_uid160253  Lactococcus_lactis_IO_1_uid192185  Lactococcus_lactis_Il1403_uid57671  Lactococcus_lactis_KF147_uid42831  Lactococcus_lactis_KLDS_4_0325_uid225028  Lactococcus_lactis_cremoris_A76_uid160937  Lactococcus_lactis_cremoris_KW2_uid219629  Lactococcus_lactis_cremoris_MG1363_uid58837  Lactococcus_lactis_cremoris_NZ9000_uid167481  Lactococcus_lactis_cremoris_SK11_uid57983  Lactococcus_lactis_cremoris_UC509_9_uid179384  Laribacter_hongkongensis_HLHK9_uid59265  Lawsonia_intracellularis_N343_uid186598  Lawsonia_intracellularis_PHE_MN1_00_uid61575  Leadbetterella_byssophila_DSM_17132_uid60161  Legionella_longbeachae_NSW150_uid46099  Legionella_pneumophila_2300_99_Alcoy_uid48801  Legionella_pneumophila_ATCC_43290_uid86885  Legionella_pneumophila_Corby_uid58733  Legionella_pneumophila_LPE509_uid193710  Legionella_pneumophila_Lens_uid58209  Legionella_pneumophila_Lorraine_uid170535  Legionella_pneumophila_Paris_uid58211  Legionella_pneumophila_Philadelphia_1_uid57609  Legionella_pneumophila_Thunder_Bay_uid206517  Legionella_pneumophila_uid170534  Leifsonia_xyli_CTCB07_uid57759  Leifsonia_xyli_cynodontis_DSM_46306_uid221294  Leisingera_methylohalidivorans_DSM_14336_uid232356  Leptolyngbya_PCC_7376_uid182928  Leptospira_biflexa_serovar_Patoc__Patoc_1__Ames__uid58511  Leptospira_biflexa_serovar_Patoc__Patoc_1__Paris__uid58993  Leptospira_borgpetersenii_serovar_Hardjo_bovis_JB197_uid58509  Leptospira_borgpetersenii_serovar_Hardjo_bovis_L550_uid58507  Leptospira_interrogans_serovar_Copenhageni_Fiocruz_L1_130_uid58065  Leptospira_interrogans_serovar_Lai_56601_uid57881  Leptospira_interrogans_serovar_Lai_IPAV_uid161957  Leptospirillum_ferriphilum_ML_04_uid175904  Leptospirillum_ferrooxidans_C2_3_uid158171  Leptothrix_cholodnii_SP_6_uid58971  Leptotrichia_buccalis_C_1013_b_uid59211  Leuconostoc_C2_uid68743  Leuconostoc_carnosum_JB16_uid176371  Leuconostoc_citreum_KM20_uid58481  Leuconostoc_gasicomitatum_LMG_18811_uid50385  Leuconostoc_gelidum_JB7_uid175682  Leuconostoc_kimchii_IMSNU_11154_uid48589  Leuconostoc_mesenteroides_ATCC_8293_uid57919  Leuconostoc_mesenteroides_J18_uid84337  Listeria_innocua_Clip11262_uid61567  Listeria_ivanovii_PAM_55_uid73473  Listeria_monocytogenes_07PF0776_uid162185  Listeria_monocytogenes_08_5923_uid43727  Listeria_monocytogenes_10403S_uid54461  Listeria_monocytogenes_ATCC_19117_uid175109  Listeria_monocytogenes_Clip80459_uid59317  Listeria_monocytogenes_EGD_e_uid61583  Listeria_monocytogenes_EGD_uid223288  Listeria_monocytogenes_FSL_R2_561_uid54441  Listeria_monocytogenes_Finland_1998_uid54443  Listeria_monocytogenes_HCC23_uid59203  Listeria_monocytogenes_J0161_uid54459  Listeria_monocytogenes_J1816_uid179734  Listeria_monocytogenes_J1_220_uid179735  Listeria_monocytogenes_L312_uid175768  Listeria_monocytogenes_La111_uid193768  Listeria_monocytogenes_M7_uid162131  Listeria_monocytogenes_N53_1_uid193767  Listeria_monocytogenes_SLCC2376_uid175111  Listeria_monocytogenes_SLCC2378_uid175105  Listeria_monocytogenes_SLCC2479_uid175108  Listeria_monocytogenes_SLCC2540_uid175106  Listeria_monocytogenes_SLCC5850_uid175110  Listeria_monocytogenes_SLCC7179_uid175107  Listeria_monocytogenes_serotype_1_2b_SLCC2755_uid52455  Listeria_monocytogenes_serotype_1_2c_SLCC2372_uid174872  Listeria_monocytogenes_serotype_4a_L99_uid161953  Listeria_monocytogenes_serotype_4b_F2365_uid57689  Listeria_monocytogenes_serotype_4b_LL195_uid182103  Listeria_monocytogenes_serotype_7_SLCC2482_uid174871  Listeria_monocytogenes_uid43671  Listeria_seeligeri_serovar_1_2b_SLCC3954_uid46215  Listeria_welshimeri_serovar_6b_SLCC5334_uid61605  Listonella_anguillarum_M3_uid217771  Lysinibacillus_sphaericus_C3_41_uid58945  Macrococcus_caseolyticus_JCSC5402_uid59003  Magnetococcus_MC_1_uid57833  Magnetospirillum_gryphiswaldense_MSR_1_uid232249  Magnetospirillum_magneticum_AMB_1_uid58527  Mahella_australiensis_50_1_BON_uid66917  Mannheimia_haemolytica_D153_uid212303  Mannheimia_haemolytica_D171_uid212304  Mannheimia_haemolytica_D174_uid212305  Mannheimia_haemolytica_M42548_uid198769  Mannheimia_haemolytica_USDA_ARS_SAM_185_uid195457  Mannheimia_haemolytica_USDA_ARS_USMARC_183_uid195458  Mannheimia_haemolytica_USMARC_2286_uid213228  Mannheimia_succiniciproducens_MBEL55E_uid58197  Maricaulis_maris_MCS10_uid58689  Marinithermus_hydrothermalis_DSM_14884_uid65783  Marinitoga_piezophila_KA3_uid81629  Marinobacter_BSs20148_uid171995  Marinobacter_adhaerens_HP15_uid162009  Marinobacter_aquaeolei_VT8_uid59419  Marinobacter_hydrocarbonoclasticus_ATCC_49840_uid162203  Marinomonas_MWYL1_uid58715  Marinomonas_mediterranea_MMB_1_uid64753  Marinomonas_posidonica_IVIA_Po_181_uid67323  Marivirga_tractuosa_DSM_4126_uid60837  Megamonas_hypermegale_uid197163  Megasphaera_elsdenii_DSM_20460_uid71135  Meiothermus_ruber_DSM_1279_uid198526  Meiothermus_ruber_DSM_1279_uid46661  Meiothermus_silvanus_DSM_9946_uid49485  Melioribacter_roseus_P3M_uid170941  Melissococcus_plutonius_ATCC_35311_uid66803  Melissococcus_plutonius_DAT561_uid89371  Mesoplasma_florum_L1_uid58055  Mesoplasma_florum_W37_uid224253  Mesorhizobium_australicum_WSM2073_uid75101  Mesorhizobium_ciceri_biovar_biserrulae_WSM1271_uid62101  Mesorhizobium_loti_MAFF303099_uid57601  Mesorhizobium_opportunistum_WSM2075_uid40861  Mesotoga_prima_MesG1_Ag_4_2_uid52599  Metallosphaera_cuprina_Ar_4_uid66329  Metallosphaera_sedula_DSM_5348_uid58717  Methanobacterium_AL_21_uid63623  Methanobacterium_MB1_uid231690  Methanobacterium_SWAN_1_uid67359  Methanobrevibacter_AbM4_uid206516  Methanobrevibacter_ruminantium_M1_uid45857  Methanobrevibacter_smithii_ATCC_35061_uid58827  Methanocaldococcus_FS406_22_uid42499  Methanocaldococcus_fervens_AG86_uid59347  Methanocaldococcus_infernus_ME_uid48803  Methanocaldococcus_jannaschii_DSM_2661_uid57713  Methanocaldococcus_vulcanius_M7_uid41131  Methanocella_arvoryzae_MRE50_uid61623  Methanocella_conradii_HZ254_uid157911  Methanocella_paludicola_SANAE_uid42887  Methanococcoides_burtonii_DSM_6242_uid58023  Methanococcus_aeolicus_Nankai_3_uid58823  Methanococcus_maripaludis_C5_uid58741  Methanococcus_maripaludis_C6_uid58947  Methanococcus_maripaludis_C7_uid58847  Methanococcus_maripaludis_S2_uid58035  Methanococcus_maripaludis_X1_uid70729  Methanococcus_vannielii_SB_uid58767  Methanococcus_voltae_A3_uid49529  Methanocorpusculum_labreanum_Z_uid58785  Methanoculleus_bourgensis_MS2_uid171377  Methanoculleus_marisnigri_JR1_uid58561  Methanohalobium_evestigatum_Z_7303_uid49857  Methanohalophilus_mahii_DSM_5219_uid47313  Methanolobus_psychrophilus_R15_uid177925  Methanomassiliicoccus_Mx1_Issoire_uid207287  Methanomethylovorans_hollandica_DSM_15978_uid184864  Methanoplanus_petrolearius_DSM_11571_uid52695  Methanopyrus_kandleri_AV19_uid57883  Methanoregula_boonei_6A8_uid58815  Methanoregula_formicicum_SMSP_uid184406  Methanosaeta_concilii_GP6_uid66207  Methanosaeta_harundinacea_6Ac_uid81199  Methanosaeta_thermophila_PT_uid58469  Methanosalsum_zhilinae_DSM_4017_uid68249  Methanosarcina_acetivorans_C2A_uid57879  Methanosarcina_barkeri_Fusaro_uid57715  Methanosarcina_mazei_Go1_uid57893  Methanosarcina_mazei_Tuc01_uid190185  Methanosphaera_stadtmanae_DSM_3091_uid58407  Methanosphaerula_palustris_E1_9c_uid59193  Methanospirillum_hungatei_JF_1_uid58181  Methanothermobacter_marburgensis_Marburg_uid51637  Methanothermobacter_thermautotrophicus_Delta_H_uid57877  Methanothermococcus_okinawensis_IH1_uid51535  Methanothermus_fervidus_DSM_2088_uid60167  Methanotorris_igneus_Kol_5_uid67321  Methylacidiphilum_infernorum_V4_uid59161  Methylibium_petroleiphilum_PM1_uid58085  Methylobacillus_flagellatus_KT_uid58049  Methylobacterium_4_46_uid58843  Methylobacterium_chloromethanicum_CM4_uid58933  Methylobacterium_extorquens_AM1_uid57605  Methylobacterium_extorquens_DM4_uid61617  Methylobacterium_extorquens_PA1_uid58821  Methylobacterium_nodulans_ORS_2060_uid59023  Methylobacterium_populi_BJ001_uid58937  Methylobacterium_radiotolerans_JCM_2831_uid58845  Methylocella_silvestris_BL2_uid59433  Methylococcus_capsulatus_Bath_uid57607  Methylocystis_SC2_uid174072  Methylomicrobium_alcaliphilum_uid77119  Methylomonas_methanica_MC09_uid67363  Methylophaga_JAM1_uid162947  Methylophaga_JAM7_uid162949  Methylotenera_301_uid49469  Methylotenera_mobilis_JLW8_uid59373  Methylovorus_MP688_uid60723  Methylovorus_glucosetrophus_SIP3_4_uid59367  Micavibrio_EPB_uid194120  Micavibrio_aeruginosavorus_ARL_13_uid73585  Microbacterium_testaceum_StLB037_uid62789  Micrococcus_luteus_NCTC_2665_uid59033  Microcoleus_PCC_7113_uid183114  Microcystis_aeruginosa_NIES_843_uid59101  Microlunatus_phosphovorus_NM_1_uid68055  Micromonospora_L5_uid45895  Micromonospora_aurantiaca_ATCC_27029_uid42501  Mobiluncus_curtisii_ATCC_43063_uid49695  Modestobacter_marinus_uid167487  Moorella_thermoacetica_ATCC_39073_uid58051  Moraxella_catarrhalis_BBH18_uid48809  Morganella_morganii_KT_uid180867  Muricauda_ruestringensis_DSM_13258_uid72479  Mycobacterium_JDM601_uid67369  Mycobacterium_JLS_uid58489  Mycobacterium_KMS_uid58491  Mycobacterium_MCS_uid58465  Mycobacterium_MOTT36Y_uid164001  Mycobacterium_VKM_Ac_1815D_uid199859  Mycobacterium_abscessus_bolletii_50594_uid205422  Mycobacterium_abscessus_uid61613  Mycobacterium_africanum_GM041182_uid68839  Mycobacterium_avium_104_uid57693  Mycobacterium_avium_paratuberculosis_K_10_uid57699  Mycobacterium_avium_paratuberculosis_MAP4_uid202426  Mycobacterium_bovis_AF2122_97_uid57695  Mycobacterium_bovis_BCG_Korea_1168P_uid189029  Mycobacterium_bovis_BCG_Mexico_uid86889  Mycobacterium_bovis_BCG_Pasteur_1173P2_uid58781  Mycobacterium_bovis_BCG_Tokyo_172_uid59281  Mycobacterium_canettii_CIPT_140010059_uid70731  Mycobacterium_canettii_CIPT_140060008_uid184829  Mycobacterium_canettii_CIPT_140070008_uid184832  Mycobacterium_canettii_CIPT_140070010_uid184828  Mycobacterium_canettii_CIPT_140070017_uid184830  Mycobacterium_chubuense_NBB4_uid168322  Mycobacterium_gilvum_PYR_GCK_uid59421  Mycobacterium_gilvum_Spyr1_uid61403  Mycobacterium_indicus_pranii_MTCC_9506_uid175523  Mycobacterium_intracellulare_ATCC_13950_uid167994  Mycobacterium_intracellulare_MOTT_02_uid89387  Mycobacterium_intracellulare_MOTT_64_uid89385  Mycobacterium_kansasii_ATCC_12478_uid55385  Mycobacterium_leprae_Br4923_uid59293  Mycobacterium_leprae_TN_uid57697  Mycobacterium_liflandii_128FXT_uid59005  Mycobacterium_marinum_M_uid59423  Mycobacterium_massiliense_GO_06_uid170732  Mycobacterium_rhodesiae_NBB3_uid75107  Mycobacterium_smegmatis_JS623_uid184820  Mycobacterium_smegmatis_MC2_155_uid171958  Mycobacterium_smegmatis_MC2_155_uid57701  Mycobacterium_tuberculosis_Beijing_NITR203_uid197218  Mycobacterium_tuberculosis_CAS_NITR204_uid202217  Mycobacterium_tuberculosis_CCDC5079_uid161943  Mycobacterium_tuberculosis_CCDC5079_uid203790  Mycobacterium_tuberculosis_CCDC5180_uid161941  Mycobacterium_tuberculosis_CDC1551_uid57775  Mycobacterium_tuberculosis_CTRI_2_uid161997  Mycobacterium_tuberculosis_EAI5_NITR206_uid202218  Mycobacterium_tuberculosis_EAI5_uid212307  Mycobacterium_tuberculosis_Erdman___ATCC_35801_uid193763  Mycobacterium_tuberculosis_F11_uid58417  Mycobacterium_tuberculosis_H37Ra_uid58853  Mycobacterium_tuberculosis_H37Rv_uid170532  Mycobacterium_tuberculosis_H37Rv_uid57777  Mycobacterium_tuberculosis_Haarlem3_NITR202_uid202216  Mycobacterium_tuberculosis_Haarlem_uid54453  Mycobacterium_tuberculosis_KZN_1435_uid59069  Mycobacterium_tuberculosis_KZN_4207_uid83619  Mycobacterium_tuberculosis_KZN_605_uid54947  Mycobacterium_tuberculosis_RGTB327_uid157907  Mycobacterium_tuberculosis_RGTB423_uid162179  Mycobacterium_tuberculosis_UT205_uid162183  Mycobacterium_tuberculosis_uid185758  Mycobacterium_ulcerans_Agy99_uid62939  Mycobacterium_vanbaalenii_PYR_1_uid58463  Mycobacterium_yongonense_05_1390_uid189649  Mycoplasma_agalactiae_PG2_uid61619  Mycoplasma_agalactiae_uid46679  Mycoplasma_arthritidis_158L3_1_uid58005  Mycoplasma_bovis_HB0801_uid168665  Mycoplasma_bovis_Hubei_1_uid68691  Mycoplasma_bovis_PG45_uid60859  Mycoplasma_capricolum_ATCC_27343_uid58525  Mycoplasma_conjunctivae_uid59325  Mycoplasma_crocodyli_MP145_uid47087  Mycoplasma_cynos_C142_uid184824  Mycoplasma_fermentans_JER_uid53543  Mycoplasma_fermentans_M64_uid62099  Mycoplasma_fermentans_PG18_uid197154  Mycoplasma_gallisepticum_CA06_2006_052_5_2P_uid172630  Mycoplasma_gallisepticum_F_uid162001  Mycoplasma_gallisepticum_NC06_2006_080_5_2P_uid172629  Mycoplasma_gallisepticum_NC08_2008_031_4_3P_uid172631  Mycoplasma_gallisepticum_NC95_13295_2_2P_uid172625  Mycoplasma_gallisepticum_NC96_1596_4_2P_uid172626  Mycoplasma_gallisepticum_NY01_2001_047_5_1P_uid172627  Mycoplasma_gallisepticum_R_high__uid161999  Mycoplasma_gallisepticum_R_low__uid57993  Mycoplasma_gallisepticum_S6_uid200523  Mycoplasma_gallisepticum_VA94_7994_1_7P_uid172624  Mycoplasma_gallisepticum_WI01_2001_043_13_2P_uid172628  Mycoplasma_genitalium_G37_uid57707  Mycoplasma_genitalium_M2288_uid173372  Mycoplasma_genitalium_M2321_uid173373  Mycoplasma_genitalium_M6282_uid173371  Mycoplasma_genitalium_M6320_uid173370  Mycoplasma_haemocanis_Illinois_uid82367  Mycoplasma_haemofelis_Langford_1_uid62461  Mycoplasma_haemofelis_Ohio2_uid162029  Mycoplasma_hominis_ATCC_23114_uid41875  Mycoplasma_hyopneumoniae_168_L_uid205052  Mycoplasma_hyopneumoniae_168_uid162053  Mycoplasma_hyopneumoniae_232_uid58205  Mycoplasma_hyopneumoniae_7422_uid212968  Mycoplasma_hyopneumoniae_7448_uid58039  Mycoplasma_hyopneumoniae_J_uid58059  Mycoplasma_hyorhinis_DBS_1050_uid228933  Mycoplasma_hyorhinis_GDL_1_uid87003  Mycoplasma_hyorhinis_HUB_1_uid51695  Mycoplasma_hyorhinis_MCLD_uid162087  Mycoplasma_hyorhinis_SK76_uid181997  Mycoplasma_leachii_99_014_6_uid162031  Mycoplasma_leachii_PG50_uid60849  Mycoplasma_mobile_163K_uid58077  Mycoplasma_mycoides_SC_Gladysdale_uid197153  Mycoplasma_mycoides_SC_PG1_uid58031  Mycoplasma_mycoides_capri_LC_95010_uid66189  Mycoplasma_ovis_Michigan_uid232247  Mycoplasma_parvum_Indiana_uid223379  Mycoplasma_penetrans_HF_2_uid57729  Mycoplasma_pneumoniae_309_uid85495  Mycoplasma_pneumoniae_FH_uid162027  Mycoplasma_pneumoniae_M129_B7_uid185759  Mycoplasma_pneumoniae_M129_uid57709  Mycoplasma_pulmonis_UAB_CTIP_uid61569  Mycoplasma_putrefaciens_KS1_uid72481  Mycoplasma_putrefaciens_Mput9231_uid198525  Mycoplasma_suis_Illinois_uid61897  Mycoplasma_suis_KI3806_uid63665  Mycoplasma_synoviae_53_uid58061  Mycoplasma_wenyonii_Massachusetts_uid170731  Myxococcus_fulvus_HW_1_uid68443  Myxococcus_stipitatus_DSM_14675_uid186549  Myxococcus_xanthus_DK_1622_uid58003  Nakamurella_multipartita_DSM_44233_uid59221  Nanoarchaeum_equitans_Kin4_M_uid58009  Natranaerobius_thermophilus_JW_NM_WN_LF_uid59001  Natrialba_magadii_ATCC_43099_uid46245  Natrinema_J7_uid171337  Natrinema_pellirubrum_DSM_15624_uid74437  Natronobacterium_gregoryi_SP2_uid74439  Natronococcus_occultus_SP4_uid184863  Natronomonas_moolapensis_8_8_11_uid190182  Natronomonas_pharaonis_DSM_2160_uid58435  Nautilia_profundicola_AmH_uid59345  Neisseria_gonorrhoeae_FA_1090_uid57611  Neisseria_gonorrhoeae_NCCP11945_uid59191  Neisseria_gonorrhoeae_TCDC_NG08107_uid161097  Neisseria_lactamica_020_06_uid60851  Neisseria_meningitidis_053442_uid58587  Neisseria_meningitidis_8013_uid161967  Neisseria_meningitidis_FAM18_uid57825  Neisseria_meningitidis_G2136_uid162085  Neisseria_meningitidis_H44_76_uid162083  Neisseria_meningitidis_M01_240149_uid162079  Neisseria_meningitidis_M01_240355_uid162075  Neisseria_meningitidis_M04_240196_uid162081  Neisseria_meningitidis_MC58_uid57817  Neisseria_meningitidis_NZ_05_33_uid162077  Neisseria_meningitidis_WUE_2594_uid162093  Neisseria_meningitidis_Z2491_uid57819  Neisseria_meningitidis_alpha14_uid61649  Neisseria_meningitidis_alpha710_uid161971  Neorickettsia_risticii_Illinois_uid58889  Neorickettsia_sennetsu_Miyayama_uid57965  Niastella_koreensis_GR20_10_uid83125  Nitratifractor_salsuginis_DSM_16511_uid62183  Nitratiruptor_SB155_2_uid58861  Nitrobacter_hamburgensis_X14_uid58293  Nitrobacter_winogradskyi_Nb_255_uid58295  Nitrosococcus_halophilus_Nc4_uid46803  Nitrosococcus_oceani_ATCC_19707_uid58403  Nitrosococcus_watsonii_C_113_uid50331  Nitrosomonas_AL212_uid55727  Nitrosomonas_Is79A3_uid68745  Nitrosomonas_europaea_ATCC_19718_uid57647  Nitrosomonas_eutropha_C91_uid58363  Nitrosopumilus_maritimus_SCM1_uid58903  Nitrosospira_multiformis_ATCC_25196_uid58361  Nocardia_brasiliensis_ATCC_700358_uid86913  Nocardia_cyriacigeorgica_GUH_2_uid89395  Nocardia_farcinica_IFM_10152_uid58203  Nocardioides_JS614_uid58149  Nocardiopsis_alba_ATCC_BAA_2165_uid174334  Nocardiopsis_dassonvillei_DSM_43111_uid49483  Nostoc_PCC_7107_uid182932  Nostoc_PCC_7120_uid57803  Nostoc_PCC_7524_uid182933  Nostoc_punctiforme_PCC_73102_uid57767  Novosphingobium_PP1Y_uid67383  Novosphingobium_aromaticivorans_DSM_12444_uid57747  Oceanimonas_GK1_uid81627  Oceanithermus_profundus_DSM_14977_uid60855  Oceanobacillus_iheyensis_HTE831_uid57867  Ochrobactrum_anthropi_ATCC_49188_uid58921  Octadecabacter_antarcticus_307_uid54701  Octadecabacter_arcticus_238_uid54699  Odoribacter_splanchnicus_DSM_20712_uid63397  Oenococcus_oeni_PSU_1_uid59417  Oligotropha_carboxidovorans_OM4_uid162135  Oligotropha_carboxidovorans_OM5_uid59155  Oligotropha_carboxidovorans_OM5_uid72795  Olsenella_uli_DSM_7084_uid51367  Onion_yellows_phytoplasma_OY_M_uid58015  Opitutus_terrae_PB90_1_uid58965  Orientia_tsutsugamushi_Boryong_uid61621  Orientia_tsutsugamushi_Ikeda_uid58869  Ornithobacterium_rhinotracheale_DSM_15997_uid168256  Oscillatoria_acuminata_PCC_6304_uid183003  Oscillatoria_nigro_viridis_PCC_7112_uid183110  Oscillibacter_valericigenes_Sjm18_20_uid73895  Owenweeksia_hongkongensis_DSM_17368_uid82951  Paenibacillus_JDR_2_uid59021  Paenibacillus_Y412MC10_uid41127  Paenibacillus_larvae_04_309_uid232355  Paenibacillus_mucilaginosus_3016_uid89377  Paenibacillus_mucilaginosus_K02_uid162117  Paenibacillus_mucilaginosus_KNP414_uid68311  Paenibacillus_polymyxa_CR1_uid231659  Paenibacillus_polymyxa_E681_uid53477  Paenibacillus_polymyxa_M1_uid162159  Paenibacillus_polymyxa_SC2_uid59583  Paenibacillus_terrae_HPL_003_uid82371  Paludibacter_propionicigenes_WB4_uid60725  Pandoraea_RB_44_uid231151  Pandoraea_pnomenusa_3kgm_uid229878  Pantoea_At_9b_uid55845  Pantoea_ananatis_AJ13355_uid162073  Pantoea_ananatis_LMG_20103_uid46807  Pantoea_ananatis_PA13_uid162181  Pantoea_ananatis_uid86861  Pantoea_vagans_C9_1_uid49871  Parabacteroides_distasonis_ATCC_8503_uid58301  Parachlamydia_acanthamoebae_UV7_uid68335  Paracoccus_aminophilus_JCM_7686_uid214795  Paracoccus_denitrificans_PD1222_uid58187  Parvibaculum_lavamentivorans_DS_1_uid58739  Parvularcula_bermudensis_HTCC2503_uid51641  Pasteurella_multocida_3480_uid161955  Pasteurella_multocida_36950_uid86887  Pasteurella_multocida_HN06_uid156881  Pasteurella_multocida_Pm70_uid57627  Pectobacterium_SCC3193_uid193707  Pectobacterium_atrosepticum_SCRI1043_uid57957  Pectobacterium_carotovorum_PC1_uid59295  Pectobacterium_carotovorum_PCC21_uid174335  Pectobacterium_wasabiae_WPP163_uid41297  Pediococcus_claussenii_ATCC_BAA_344_uid81103  Pediococcus_pentosaceus_ATCC_25745_uid57981  Pediococcus_pentosaceus_SL4_uid227215  Pedobacter_heparinus_DSM_2366_uid59111  Pedobacter_saltans_DSM_12145_uid61349  Pelagibacterium_halotolerans_B2_uid74393  Pelobacter_carbinolicus_DSM_2380_uid58241  Pelobacter_propionicus_DSM_2379_uid58255  Pelodictyon_phaeoclathratiforme_BU_1_uid58173  Pelotomaculum_thermopropionicum_SI_uid58877  Persephonella_marina_EX_H1_uid58119  Persicivirga_dokdonensis_DSW_6_uid186842  Petrotoga_mobilis_SJ95_uid58747  Phaeobacter_gallaeciensis_DSM_17395_uid54717  Phaeobacter_gallaeciensis_DSM_26640_uid232357  Phaeobacter_gallaeciensis_uid54715  Phenylobacterium_zucineum_HLK1_uid58959  Photobacterium_profundum_SS9_uid62923  Photorhabdus_asymbiotica_ATCC_43949_uid59243  Photorhabdus_luminescens_laumondii_TTO1_uid61593  Phycisphaera_mikurensis_NBRC_102666_uid157331  Picrophilus_torridus_DSM_9790_uid58041  Pirellula_staleyi_DSM_6068_uid43209  Planctomyces_brasiliensis_DSM_5305_uid60583  Planctomyces_limnophilus_DSM_3776_uid48643  Plautia_stali_symbiont_uid65033  Pleurocapsa_PCC_7327_uid183006  Polaribacter_MED152_uid54207  Polaromonas_JS666_uid58207  Polaromonas_naphthalenivorans_CJ2_uid58273  Polymorphum_gilvum_SL003B_26A1_uid65447  Polynucleobacter_necessarius_STIR1_uid58967  Polynucleobacter_necessarius_asymbioticus_QLW_P1DMWA_1_uid58611  Porphyromonas_asaccharolytica_DSM_20707_uid66603  Porphyromonas_gingivalis_ATCC_33277_uid58879  Porphyromonas_gingivalis_TDC60_uid67407  Porphyromonas_gingivalis_W83_uid57641  Prevotella_dentalis_DSM_3688_uid184818  Prevotella_denticola_F0289_uid65091  Prevotella_intermedia_17_uid163151  Prevotella_melaninogenica_ATCC_25845_uid51377  Prevotella_oral_taxon_299_F0039_uid45899  Prevotella_ruminicola_23_uid47507  Prochlorococcus_marinus_AS9601_uid58307  Prochlorococcus_marinus_CCMP1375_uid57995  Prochlorococcus_marinus_MIT_9211_uid58309  Prochlorococcus_marinus_MIT_9215_uid58819  Prochlorococcus_marinus_MIT_9301_uid58437  Prochlorococcus_marinus_MIT_9303_uid58305  Prochlorococcus_marinus_MIT_9312_uid58357  Prochlorococcus_marinus_MIT_9313_uid57773  Prochlorococcus_marinus_MIT_9515_uid58313  Prochlorococcus_marinus_NATL1A_uid58423  Prochlorococcus_marinus_NATL2A_uid58359  Prochlorococcus_marinus_pastoris_CCMP1986_uid57761  Propionibacterium_acidipropionici_ATCC_4875_uid179069  Propionibacterium_acnes_266_uid162059  Propionibacterium_acnes_6609_uid162137  Propionibacterium_acnes_ATCC_11828_uid162177  Propionibacterium_acnes_C1_uid176501  Propionibacterium_acnes_HL096PA1_uid198524  Propionibacterium_acnes_KPA171202_uid58101  Propionibacterium_acnes_SK137_uid48071  Propionibacterium_acnes_TypeIA2_P_acn17_uid80735  Propionibacterium_acnes_TypeIA2_P_acn31_uid80733  Propionibacterium_acnes_TypeIA2_P_acn33_uid80745  Propionibacterium_avidum_44067_uid197361  Propionibacterium_freudenreichii_shermanii_CIRM_BIA1_uid49535  Propionibacterium_propionicum_F0230a_uid170533  Prosthecochloris_aestuarii_DSM_271_uid58151  Proteus_mirabilis_BB2000_uid214430  Proteus_mirabilis_HI4320_uid61599  Providencia_stuartii_MRSN_2154_uid162193  Pseudanabaena_PCC_7367_uid183004  Pseudoalteromonas_SM9913_uid61247  Pseudoalteromonas_atlantica_T6c_uid58283  Pseudoalteromonas_haloplanktis_TAC125_uid58431  Pseudogulbenkiania_NH8B_uid73423  Pseudomonas_ND6_uid167583  Pseudomonas_TKP_uid232248  Pseudomonas_VLB120_uid226717  Pseudomonas_aeruginosa_B136_33_uid196598  Pseudomonas_aeruginosa_DK2_uid168996  Pseudomonas_aeruginosa_LES431_uid232245  Pseudomonas_aeruginosa_LESB58_uid59275  Pseudomonas_aeruginosa_M18_uid162089  Pseudomonas_aeruginosa_MTB_uid231150  Pseudomonas_aeruginosa_NCGM2_S1_uid162173  Pseudomonas_aeruginosa_PA1R_uid228932  Pseudomonas_aeruginosa_PA1_uid228931  Pseudomonas_aeruginosa_PA7_uid58627  Pseudomonas_aeruginosa_PAO1_VE13_uid225027  Pseudomonas_aeruginosa_PAO1_VE2_uid225026  Pseudomonas_aeruginosa_PAO1_uid57945  Pseudomonas_aeruginosa_PAO581_uid219357  Pseudomonas_aeruginosa_RP73_uid209328  Pseudomonas_aeruginosa_SCV20265_uid232358  Pseudomonas_aeruginosa_UCBPP_PA14_uid57977  Pseudomonas_aeruginosa_c7447m_uid219358  Pseudomonas_brassicacearum_NFM421_uid66303  Pseudomonas_denitrificans_ATCC_13867_uid195459  Pseudomonas_entomophila_L48_uid58639  Pseudomonas_fluorescens_A506_uid165185  Pseudomonas_fluorescens_CHA0_uid203393  Pseudomonas_fluorescens_F113_uid87037  Pseudomonas_fluorescens_Pf0_1_uid57591  Pseudomonas_fluorescens_Pf_5_uid57937  Pseudomonas_fluorescens_R124_uid182045  Pseudomonas_fluorescens_SBW25_uid158693  Pseudomonas_fulva_12_X_uid67351  Pseudomonas_mendocina_NK_01_uid66299  Pseudomonas_mendocina_ymp_uid58723  Pseudomonas_monteilii_SB3078_uid232252  Pseudomonas_monteilii_SB3101_uid232253  Pseudomonas_poae_RE_1_1_14_uid188480  Pseudomonas_putida_BIRD_1_uid162055  Pseudomonas_putida_DOT_T1E_uid171260  Pseudomonas_putida_F1_uid58355  Pseudomonas_putida_GB_1_uid58735  Pseudomonas_putida_H8234_uid208673  Pseudomonas_putida_HB3267_uid184078  Pseudomonas_putida_KT2440_uid57843  Pseudomonas_putida_NBRC_14164_uid208670  Pseudomonas_putida_S16_uid68747  Pseudomonas_putida_UW4_uid182733  Pseudomonas_putida_W619_uid58651  Pseudomonas_resinovorans_NBRC_106553_uid208671  Pseudomonas_stutzeri_A1501_uid58641  Pseudomonas_stutzeri_ATCC_17588___LMG_11199_uid68749  Pseudomonas_stutzeri_CCUG_29243_uid168379  Pseudomonas_stutzeri_DSM_10701_uid170940  Pseudomonas_stutzeri_DSM_4166_uid162113  Pseudomonas_stutzeri_RCH2_uid184342  Pseudomonas_syringae_B728a_uid57931  Pseudomonas_syringae_phaseolicola_1448A_uid58099  Pseudomonas_syringae_tomato_DC3000_uid57967  Pseudonocardia_dioxanivorans_CB1190_uid65087  Pseudovibrio_FO_BEG1_uid82373  Pseudoxanthomonas_spadix_BD_a59_uid75113  Pseudoxanthomonas_suwonensis_11_1_uid62105  Psychrobacter_G_uid210641  Psychrobacter_PRwf_1_uid58459  Psychrobacter_arcticus_273_4_uid58021  Psychrobacter_cryohalolentis_K5_uid58373  Psychroflexus_torquis_ATCC_700755_uid54205  Psychromonas_CNPT3_uid54249  Psychromonas_ingrahamii_37_uid58521  Pusillimonas_T7_7_uid66391  Pyrobaculum_1860_uid82379  Pyrobaculum_aerophilum_IM2_uid57727  Pyrobaculum_arsenaticum_DSM_13514_uid58409  Pyrobaculum_calidifontis_JCM_11548_uid58787  Pyrobaculum_islandicum_DSM_4184_uid58635  Pyrobaculum_neutrophilum_V24Sta_uid58421  Pyrobaculum_oguniense_TE7_uid84411  Pyrococcus_NA2_uid66551  Pyrococcus_ST04_uid167261  Pyrococcus_abyssi_GE5_uid62903  Pyrococcus_furiosus_COM1_uid169620  Pyrococcus_furiosus_DSM_3638_uid57873  Pyrococcus_horikoshii_OT3_uid57753  Pyrococcus_yayanosii_CH1_uid68281  Pyrolobus_fumarii_1A_uid73415  Rahnella_Y9602_uid62715  Rahnella_aquatilis_CIP_78_65___ATCC_33071_uid86855  Rahnella_aquatilis_HX2_uid158049  Ralstonia_eutropha_H16_uid62925  Ralstonia_eutropha_JMP134_uid58047  Ralstonia_pickettii_12D_uid58859  Ralstonia_pickettii_12J_uid58737  Ralstonia_pickettii_DTP0602_uid222229  Ralstonia_solanacearum_CFBP2957_uid50545  Ralstonia_solanacearum_CMR15_uid227773  Ralstonia_solanacearum_FQY_4_f_uid194089  Ralstonia_solanacearum_GMI1000_uid57593  Ralstonia_solanacearum_PSI07_uid50539  Ralstonia_solanacearum_Po82_uid162133  Ramlibacter_tataouinensis_TTB310_uid68279  Raoultella_ornithinolytica_B6_uid198431  Renibacterium_salmoninarum_ATCC_33209_uid58899  Rhizobium_IRBG74_uid222820  Rhizobium_NGR234_uid59081  Rhizobium_etli_CFN_42_uid58377  Rhizobium_etli_CIAT_652_uid59115  Rhizobium_etli_bv__mimosae_Mim1_uid213896  Rhizobium_leguminosarum_bv__trifolii_WSM1325_uid58991  Rhizobium_leguminosarum_bv__trifolii_WSM2304_uid58997  Rhizobium_leguminosarum_bv__viciae_3841_uid57955  Rhizobium_tropici_CIAT_899_uid185179  Rhodanobacter_2APBS1_uid74431  Rhodobacter_capsulatus_SB_1003_uid47509  Rhodobacter_sphaeroides_2_4_1_uid57653  Rhodobacter_sphaeroides_ATCC_17025_uid58451  Rhodobacter_sphaeroides_ATCC_17029_uid58449  Rhodobacter_sphaeroides_KD131_uid59277  Rhodococcus_equi_103S_uid60171  Rhodococcus_erythropolis_CCM2595_uid216088  Rhodococcus_erythropolis_PR4_uid59019  Rhodococcus_jostii_RHA1_uid58325  Rhodococcus_opacus_B4_uid13791  Rhodococcus_pyridinivorans_SB3094_uid232359  Rhodoferax_ferrireducens_T118_uid58353  Rhodomicrobium_vannielii_ATCC_17100_uid43247  Rhodopirellula_baltica_SH_1_uid61589  Rhodopseudomonas_palustris_BisA53_uid58445  Rhodopseudomonas_palustris_BisB18_uid58443  Rhodopseudomonas_palustris_BisB5_uid58441  Rhodopseudomonas_palustris_CGA009_uid62901  Rhodopseudomonas_palustris_DX_1_uid43327  Rhodopseudomonas_palustris_HaA2_uid58439  Rhodopseudomonas_palustris_TIE_1_uid58995  Rhodospirillum_centenum_SW_uid58805  Rhodospirillum_photometricum_uid159003  Rhodospirillum_rubrum_ATCC_11170_uid57655  Rhodospirillum_rubrum_F11_uid162149  Rhodothermus_marinus_DSM_4252_uid41729  Rhodothermus_marinus_SG0_5JP17_172_uid72767  Rickettsia_africae_ESF_5_uid58799  Rickettsia_akari_Hartford_uid58161  Rickettsia_australis_Cutlack_uid158039  Rickettsia_bellii_OSU_85_389_uid58681  Rickettsia_bellii_RML369_C_uid58405  Rickettsia_canadensis_CA410_uid88063  Rickettsia_canadensis_McKiel_uid58159  Rickettsia_conorii_Malish_7_uid57633  Rickettsia_felis_URRWXCal2_uid58331  Rickettsia_heilongjiangensis_054_uid70839  Rickettsia_japonica_YH_uid73963  Rickettsia_massiliae_AZT80_uid86751  Rickettsia_massiliae_MTU5_uid58801  Rickettsia_montanensis_OSU_85_930_uid158043  Rickettsia_parkeri_Portsmouth_uid158045  Rickettsia_peacockii_Rustic_uid59301  Rickettsia_philipii_364D_uid89383  Rickettsia_prowazekii_Breinl_uid196851  Rickettsia_prowazekii_BuV67_CWPP_uid158063  Rickettsia_prowazekii_Chernikova_uid158053  Rickettsia_prowazekii_Dachau_uid158057  Rickettsia_prowazekii_GvV257_uid158051  Rickettsia_prowazekii_Katsinyian_uid158055  Rickettsia_prowazekii_Madrid_E_uid61565  Rickettsia_prowazekii_NMRC_Madrid_E_uid196850  Rickettsia_prowazekii_Rp22_uid161945  Rickettsia_prowazekii_RpGvF24_uid158065  Rickettsia_rhipicephali_3_7_female6_CWPP_uid156977  Rickettsia_rickettsii_Arizona_uid86655  Rickettsia_rickettsii_Brazil_uid88069  Rickettsia_rickettsii_Colombia_uid86653  Rickettsia_rickettsii_Hauke_uid86659  Rickettsia_rickettsii_Hino_uid86657  Rickettsia_rickettsii_Hlp_2_uid88067  Rickettsia_rickettsii_Iowa_uid58961  Rickettsia_rickettsii__Sheila_Smith__uid58027  Rickettsia_slovaca_13_B_uid82369  Rickettsia_slovaca_D_CWPP_uid158159  Rickettsia_typhi_B9991CWPP_uid158357  Rickettsia_typhi_TH1527_uid158161  Rickettsia_typhi_Wilmington_uid58063  Riemerella_anatipestifer_ATCC_11845___DSM_15868_uid159857  Riemerella_anatipestifer_ATCC_11845___DSM_15868_uid60727  Riemerella_anatipestifer_RA_CH_1_uid175469  Riemerella_anatipestifer_RA_CH_2_uid186548  Riemerella_anatipestifer_RA_GD_uid162013  Rivularia_PCC_7116_uid182929  Robiginitalea_biformata_HTCC2501_uid58285  Roseburia_hominis_A2_183_uid73419  Roseburia_intestinalis_XB6B4_uid197179  Roseburia_intestinalis_uid197164  Roseiflexus_RS_1_uid58523  Roseiflexus_castenholzii_DSM_13941_uid58287  Roseobacter_denitrificans_OCh_114_uid58597  Roseobacter_litoralis_Och_149_uid54719  Rothia_dentocariosa_ATCC_17931_uid49331  Rothia_mucilaginosa_uid43093  Rubrivivax_gelatinosus_IL144_uid158163  Rubrobacter_xylanophilus_DSM_9941_uid58057  Ruegeria_TM1040_uid58193  Ruegeria_pomeroyi_DSS_3_uid57863  Ruminococcus_albus_7_uid51721  Ruminococcus_bromii_uid197158  Ruminococcus_champanellensis_18P13_uid197169  Ruminococcus_uid197156  Runella_slithyformis_DSM_19594_uid68317  Saccharomonospora_viridis_DSM_43017_uid59055  Saccharophagus_degradans_2_40_uid57921  Saccharopolyspora_erythraea_NRRL_2338_uid62947  Saccharothrix_espanaensis_DSM_44229_uid184826  Salinarchaeum_laminariae_Harcht_Bsk1_uid207001  Salinibacter_ruber_DSM_13855_uid58513  Salinibacter_ruber_M8_uid47323  Salinispora_arenicola_CNS_205_uid58659  Salinispora_tropica_CNB_440_uid58565  Salmonella_bongori_NCTC_12419_uid70155  Salmonella_bongori_Sbon_167_uid213088  Salmonella_enterica_Serovar_Cubana_CFSAN002050_uid212973  Salmonella_enterica_Serovar_Heidelberg_CFSAN002069_uid212974  Salmonella_enterica_Serovar_Typhimurium_var__5__CFSAN001921_uid212972  Salmonella_enterica_arizonae_serovar_62_z4_z23__uid58191  Salmonella_enterica_serovar_4_5_12_i__08_1736_uid212969  Salmonella_enterica_serovar_Agona_24249_uid230614  Salmonella_enterica_serovar_Agona_SL483_uid59431  Salmonella_enterica_serovar_Bareilly_CFSAN000189_uid212971  Salmonella_enterica_serovar_Bovismorbificans_3114_uid218006  Salmonella_enterica_serovar_Choleraesuis_SC_B67_uid58017  Salmonella_enterica_serovar_Dublin_CT_02021853_uid58917  Salmonella_enterica_serovar_Enteritidis_P125109_uid59247  Salmonella_enterica_serovar_Gallinarum_287_91_uid59249  Salmonella_enterica_serovar_Gallinarum_Pullorum_CDC1983_67_uid217770  Salmonella_enterica_serovar_Gallinarum_pullorum_RKS5078_uid87035  Salmonella_enterica_serovar_Heidelberg_41578_uid212970  Salmonella_enterica_serovar_Heidelberg_B182_uid162195  Salmonella_enterica_serovar_Heidelberg_SL476_uid58973  Salmonella_enterica_serovar_Javiana_CFSAN001992_uid190101  Salmonella_enterica_serovar_Newport_SL254_uid58831  Salmonella_enterica_serovar_Newport_USMARC_S3124_1_uid213895  Salmonella_enterica_serovar_Paratyphi_A_AKU_12601_uid59269  Salmonella_enterica_serovar_Paratyphi_A_ATCC_9150_uid58201  Salmonella_enterica_serovar_Paratyphi_B_SPB7_uid59097  Salmonella_enterica_serovar_Paratyphi_C_RKS4594_uid59063  Salmonella_enterica_serovar_Pullorum_S06004_uid214431  Salmonella_enterica_serovar_Schwarzengrund_CVM19633_uid58915  Salmonella_enterica_serovar_Thompson_RM6836_uid222802  Salmonella_enterica_serovar_Typhi_CT18_uid57793  Salmonella_enterica_serovar_Typhi_P_stx_12_uid87001  Salmonella_enterica_serovar_Typhi_Ty21a_uid201427  Salmonella_enterica_serovar_Typhi_Ty2_uid57973  Salmonella_enterica_serovar_Typhimurium_14028S_uid86059  Salmonella_enterica_serovar_Typhimurium_798_uid158047  Salmonella_enterica_serovar_Typhimurium_D23580_uid86061  Salmonella_enterica_serovar_Typhimurium_DT2_uid222818  Salmonella_enterica_serovar_Typhimurium_LT2_uid57799  Salmonella_enterica_serovar_Typhimurium_SL1344_uid86645  Salmonella_enterica_serovar_Typhimurium_ST4_74_uid84393  Salmonella_enterica_serovar_Typhimurium_T000240_uid84397  Salmonella_enterica_serovar_Typhimurium_U288_uid198746  Salmonella_enterica_serovar_Typhimurium_UK_1_uid87049  Salmonella_enterica_serovar_Weltevreden_2007_60_3289_1_uid178014  Salmonella_typhimurium_DT104_uid223287  Sanguibacter_keddieii_DSM_10542_uid40845  Saprospira_grandis_Lewin_uid89375  Sebaldella_termitidis_ATCC_33386_uid41865  Segniliparus_rotundus_DSM_44985_uid49049  Selenomonas_ruminantium_lactilytica_TAM6421_uid157247  Selenomonas_sputigena_ATCC_35185_uid55329  Serratia_AS12_uid67315  Serratia_AS13_uid162065  Serratia_ATCC_39006_uid218470  Serratia_liquefaciens_ATCC_27592_uid212306  Serratia_marcescens_FGI94_uid185180  Serratia_marcescens_WW4_uid188478  Serratia_odorifera_4Rx13_uid42253  Serratia_plymuthica_AS9_uid67313  Serratia_plymuthica_S13_uid210642  Serratia_proteamaculans_568_uid58725  Serratia_symbiotica__Cinara_cedri__uid82363  Shewanella_ANA_3_uid58347  Shewanella_MR_4_uid58345  Shewanella_MR_7_uid58343  Shewanella_W3_18_1_uid58341  Shewanella_amazonensis_SB2B_uid58257  Shewanella_baltica_BA175_uid52601  Shewanella_baltica_OS117_uid162025  Shewanella_baltica_OS155_uid58259  Shewanella_baltica_OS185_uid58743  Shewanella_baltica_OS195_uid58261  Shewanella_baltica_OS223_uid58775  Shewanella_baltica_OS678_uid50553  Shewanella_denitrificans_OS217_uid58263  Shewanella_frigidimarina_NCIMB_400_uid58265  Shewanella_halifaxensis_HAW_EB4_uid59007  Shewanella_loihica_PV_4_uid58349  Shewanella_oneidensis_MR_1_uid57949  Shewanella_pealeana_ATCC_700345_uid58705  Shewanella_piezotolerans_WP3_uid58745  Shewanella_putrefaciens_200_uid161927  Shewanella_putrefaciens_CN_32_uid58267  Shewanella_sediminis_HAW_EB3_uid58835  Shewanella_violacea_DSS12_uid47085  Shewanella_woodyi_ATCC_51908_uid58721  Shigella_boydii_CDC_3083_94_uid58415  Shigella_boydii_Sb227_uid58215  Shigella_dysenteriae_1617_uid229875  Shigella_dysenteriae_Sd197_uid58213  Shigella_flexneri_2002017_uid159233  Shigella_flexneri_2a_2457T_uid57991  Shigella_flexneri_2a_301_uid62907  Shigella_flexneri_5_8401_uid58583  Shigella_sonnei_53G_uid84383  Shigella_sonnei_Ss046_uid58217  Sideroxydans_lithotrophicus_ES_1_uid46801  Simiduia_agarivorans_SA1_uid177713  Simkania_negevensis_Z_uid68451  Singulisphaera_acidiphila_DSM_18658_uid81777  Sinorhizobium_fredii_HH103_uid86865  Sinorhizobium_fredii_USDA_257_uid168059  Sinorhizobium_medicae_WSM419_uid58549  Sinorhizobium_meliloti_1021_uid57603  Sinorhizobium_meliloti_2011_uid193772  Sinorhizobium_meliloti_AK83_uid52607  Sinorhizobium_meliloti_BL225C_uid52605  Sinorhizobium_meliloti_GR4_uid184823  Sinorhizobium_meliloti_Rm41_uid176372  Sinorhizobium_meliloti_SM11_uid159685  Slackia_heliotrinireducens_DSM_20476_uid59051  Sodalis_glossinidius__morsitans__uid58553  Solibacillus_silvestris_StLB046_uid168516  Solitalea_canadensis_DSM_3403_uid81783  Sorangium_cellulosum_So0157_2_uid210741  Sorangium_cellulosum__So_ce_56__uid61629  Sphaerobacter_thermophilus_DSM_20745_uid41997  Sphaerochaeta_pleomorpha_Grapes_uid82365  Sphingobacterium_21_uid64755  Sphingobium_SYK_6_uid73353  Sphingobium_chlorophenolicum_L_1_uid52597  Sphingobium_japonicum_UT26S_uid47077  Sphingomonas_MM_1_uid193771  Sphingomonas_wittichii_RW1_uid58691  Sphingopyxis_alaskensis_RB2256_uid58351  Spiribacter_UAH_SP71_uid226111  Spirochaeta_Buddy_uid63633  Spirochaeta_L21_RPul_D2_uid231658  Spirochaeta_africana_DSM_8902_uid81779  Spirochaeta_caldaria_DSM_7334_uid68753  Spirochaeta_coccoides_DSM_17374_uid66331  Spirochaeta_smaragdinae_DSM_11293_uid51369  Spirochaeta_thermophila_DSM_6192_uid53037  Spirochaeta_thermophila_DSM_6578_uid162041  Spiroplasma_apis_B31_uid230613  Spiroplasma_chrysopicola_DF_1_uid205053  Spiroplasma_diminutum_CUAS_1_uid212976  Spiroplasma_syrphidicola_EA_1_uid205054  Spiroplasma_taiwanense_CT_1_uid212975  Spirosoma_linguale_DSM_74_uid43413  Stackebrandtia_nassauensis_DSM_44728_uid46663  Stanieria_cyanosphaera_PCC_7437_uid183115  Staphylococcus_aureus_04_02981_uid161969  Staphylococcus_aureus_08BA02176_uid175257  Staphylococcus_aureus_11819_97_uid159981  Staphylococcus_aureus_55_2053_uid55909  Staphylococcus_aureus_6850_uid217772  Staphylococcus_aureus_71193_uid162141  Staphylococcus_aureus_Bmb9393_uid210640  Staphylococcus_aureus_CC45_uid209174  Staphylococcus_aureus_CN1_uid217769  Staphylococcus_aureus_COL_uid57797  Staphylococcus_aureus_ECT_R_2_uid159389  Staphylococcus_aureus_ED133_uid159689  Staphylococcus_aureus_ED98_uid41455  Staphylococcus_aureus_HO_5096_0412_uid162163  Staphylococcus_aureus_JH1_uid58457  Staphylococcus_aureus_JH9_uid58455  Staphylococcus_aureus_JKD6008_uid159855  Staphylococcus_aureus_JKD6159_uid159691  Staphylococcus_aureus_LGA251_uid159391  Staphylococcus_aureus_M013_uid88065  Staphylococcus_aureus_M1_uid197263  Staphylococcus_aureus_MRSA252_uid57839  Staphylococcus_aureus_MSHR1132_uid89393  Staphylococcus_aureus_MSSA476_uid57841  Staphylococcus_aureus_MW2_uid57903  Staphylococcus_aureus_Mu3_uid58817  Staphylococcus_aureus_Mu50_uid57835  Staphylococcus_aureus_N315_uid57837  Staphylococcus_aureus_NCTC_8325_uid57795  Staphylococcus_aureus_Newman_uid58839  Staphylococcus_aureus_RF122_uid57661  Staphylococcus_aureus_SA40_uid221289  Staphylococcus_aureus_SA957_uid221288  Staphylococcus_aureus_ST228_10388_uid193754  Staphylococcus_aureus_ST228_10497_uid193755  Staphylococcus_aureus_ST228_15532_uid193756  Staphylococcus_aureus_ST228_16035_uid193757  Staphylococcus_aureus_ST228_18412_uid193760  Staphylococcus_aureus_ST228_18583_uid193761  Staphylococcus_aureus_ST398_uid159247  Staphylococcus_aureus_T0131_uid159861  Staphylococcus_aureus_TCH60_uid159859  Staphylococcus_aureus_TW20_uid159241  Staphylococcus_aureus_USA300_FPR3757_uid58555  Staphylococcus_aureus_USA300_TCH1516_uid58925  Staphylococcus_aureus_VC40_uid88071  Staphylococcus_aureus_Z172_uid225604  Staphylococcus_aureus_uid193758  Staphylococcus_aureus_uid193759  Staphylococcus_carnosus_TM300_uid59401  Staphylococcus_epidermidis_ATCC_12228_uid57861  Staphylococcus_epidermidis_RP62A_uid57663  Staphylococcus_haemolyticus_JCSC1435_uid62919  Staphylococcus_lugdunensis_HKU09_01_uid46233  Staphylococcus_lugdunensis_N920143_uid162143  Staphylococcus_pasteuri_SP1_uid226267  Staphylococcus_pseudintermedius_ED99_uid162109  Staphylococcus_pseudintermedius_HKU10_03_uid62125  Staphylococcus_saprophyticus_ATCC_15305_uid58411  Staphylococcus_warneri_SG1_uid187059  Staphylothermus_hellenicus_DSM_12710_uid45893  Staphylothermus_marinus_F1_uid58719  Starkeya_novella_DSM_506_uid48815  Stenotrophomonas_maltophilia_D457_uid162199  Stenotrophomonas_maltophilia_JV3_uid72473  Stenotrophomonas_maltophilia_K279a_uid61647  Stenotrophomonas_maltophilia_R551_3_uid58657  Stigmatella_aurantiaca_DW4_3_1_uid158509  Strawberry_lethal_yellows_phytoplasma__CPA__NZSb11_uid203392  Streptobacillus_moniliformis_DSM_12112_uid41863  Streptococcus_I_G2_uid224251  Streptococcus_I_P16_uid224252  Streptococcus_agalactiae_09mas018883_uid208674  Streptococcus_agalactiae_2603V_R_uid57943  Streptococcus_agalactiae_2_22_uid202215  Streptococcus_agalactiae_A909_uid57935  Streptococcus_agalactiae_GD201008_001_uid175780  Streptococcus_agalactiae_ILRI005_uid208676  Streptococcus_agalactiae_ILRI112_uid208675  Streptococcus_agalactiae_NEM316_uid61585  Streptococcus_agalactiae_SA20_06_uid178722  Streptococcus_anginosus_C1051_uid218003  Streptococcus_anginosus_C238_uid218004  Streptococcus_constellatus_pharyngis_C1050_uid218002  Streptococcus_constellatus_pharyngis_C232_uid217998  Streptococcus_constellatus_pharyngis_C818_uid218001  Streptococcus_dysgalactiae_equisimilis_167_uid222822  Streptococcus_dysgalactiae_equisimilis_AC_2713_uid178644  Streptococcus_dysgalactiae_equisimilis_ATCC_12394_uid161979  Streptococcus_dysgalactiae_equisimilis_GGS_124_uid59103  Streptococcus_dysgalactiae_equisimilis_RE378_uid176684  Streptococcus_equi_4047_uid59259  Streptococcus_equi_zooepidemicus_ATCC_35246_uid162155  Streptococcus_equi_zooepidemicus_MGCS10565_uid59263  Streptococcus_equi_zooepidemicus_uid59261  Streptococcus_gallolyticus_ATCC_43143_uid162103  Streptococcus_gallolyticus_ATCC_BAA_2069_uid63617  Streptococcus_gallolyticus_UCN34_uid46061  Streptococcus_gordonii_Challis_substr__CH1_uid57667  Streptococcus_infantarius_CJ18_uid87033  Streptococcus_iniae_SF1_uid206041  Streptococcus_intermedius_B196_uid218000  Streptococcus_intermedius_C270_uid217999  Streptococcus_intermedius_JTH08_uid168614  Streptococcus_lutetiensis_033_uid213397  Streptococcus_macedonicus_ACA_DC_198_uid81631  Streptococcus_mitis_B6_uid46097  Streptococcus_mutans_GS_5_uid169223  Streptococcus_mutans_LJ23_uid162197  Streptococcus_mutans_NN2025_uid46353  Streptococcus_mutans_UA159_uid57947  Streptococcus_oligofermentans_AS_1_3089_uid201429  Streptococcus_oralis_Uo5_uid65449  Streptococcus_parasanguinis_ATCC_15912_uid49313  Streptococcus_parasanguinis_FW213_uid163997  Streptococcus_parauberis_KCTC_11537_uid67355  Streptococcus_pasteurianus_ATCC_43144_uid68019  Streptococcus_pneumoniae_670_6B_uid52533  Streptococcus_pneumoniae_70585_uid59125  Streptococcus_pneumoniae_A026_uid226114  Streptococcus_pneumoniae_AP200_uid52453  Streptococcus_pneumoniae_ATCC_700669_uid59287  Streptococcus_pneumoniae_CGSP14_uid59181  Streptococcus_pneumoniae_D39_uid58581  Streptococcus_pneumoniae_G54_uid59167  Streptococcus_pneumoniae_Hungary19A_6_uid59117  Streptococcus_pneumoniae_INV104_uid162039  Streptococcus_pneumoniae_INV200_uid162035  Streptococcus_pneumoniae_JJA_uid59121  Streptococcus_pneumoniae_OXC141_uid162037  Streptococcus_pneumoniae_P1031_uid59123  Streptococcus_pneumoniae_R6_uid57859  Streptococcus_pneumoniae_SPN034156_uid197185  Streptococcus_pneumoniae_SPN034183_uid197186  Streptococcus_pneumoniae_SPN994038_uid197187  Streptococcus_pneumoniae_SPN994039_uid197188  Streptococcus_pneumoniae_SPNA45_uid174986  Streptococcus_pneumoniae_ST556_uid162191  Streptococcus_pneumoniae_TCH8431_19A_uid49735  Streptococcus_pneumoniae_TIGR4_uid57857  Streptococcus_pneumoniae_Taiwan19F_14_uid59119  Streptococcus_pneumoniae_gamPNI0373_uid175861  Streptococcus_pseudopneumoniae_IS7493_uid71153  Streptococcus_pyogenes_A20_uid178106  Streptococcus_pyogenes_Alab49_uid162171  Streptococcus_pyogenes_HSC5_uid212978  Streptococcus_pyogenes_M1_476_uid193766  Streptococcus_pyogenes_M1_GAS_uid57845  Streptococcus_pyogenes_MGAS10270_uid58571  Streptococcus_pyogenes_MGAS10394_uid58105  Streptococcus_pyogenes_MGAS10750_uid58575  Streptococcus_pyogenes_MGAS15252_uid158037  Streptococcus_pyogenes_MGAS1882_uid158061  Streptococcus_pyogenes_MGAS2096_uid58573  Streptococcus_pyogenes_MGAS315_uid57911  Streptococcus_pyogenes_MGAS5005_uid58337  Streptococcus_pyogenes_MGAS6180_uid58335  Streptococcus_pyogenes_MGAS8232_uid57871  Streptococcus_pyogenes_MGAS9429_uid58569  Streptococcus_pyogenes_Manfredo_uid57847  Streptococcus_pyogenes_NZ131_uid59035  Streptococcus_pyogenes_SSI_1_uid57895  Streptococcus_salivarius_57_I_uid162151  Streptococcus_salivarius_CCHSS3_uid70481  Streptococcus_salivarius_JIM8777_uid162145  Streptococcus_sanguinis_SK36_uid58381  Streptococcus_suis_05ZYH33_uid58663  Streptococcus_suis_98HAH33_uid58665  Streptococcus_suis_A7_uid162111  Streptococcus_suis_BM407_uid59321  Streptococcus_suis_D12_uid162127  Streptococcus_suis_D9_uid162125  Streptococcus_suis_GZ1_uid161937  Streptococcus_suis_JS14_uid162095  Streptococcus_suis_P1_7_uid32235  Streptococcus_suis_S735_uid174333  Streptococcus_suis_SC070731_uid193769  Streptococcus_suis_SC84_uid59323  Streptococcus_suis_SS12_uid162123  Streptococcus_suis_ST1_uid167482  Streptococcus_suis_ST3_uid66327  Streptococcus_suis_T15_uid226112  Streptococcus_suis_TL13_uid203123  Streptococcus_suis_YB51_uid222230  Streptococcus_thermophilus_CNRZ1066_uid58221  Streptococcus_thermophilus_JIM_8232_uid162157  Streptococcus_thermophilus_LMD_9_uid58327  Streptococcus_thermophilus_LMG_18311_uid58219  Streptococcus_thermophilus_MN_ZLW_002_uid166827  Streptococcus_thermophilus_ND03_uid162015  Streptococcus_uberis_0140J_uid57959  Streptomyces_PAMC26508_uid197217  Streptomyces_SirexAA_E_uid72627  Streptomyces_albus_J1074_uid196849  Streptomyces_avermitilis_MA_4680_uid57739  Streptomyces_bingchenggensis_BCW_1_uid82931  Streptomyces_cattleya_NRRL_8057___DSM_46488_uid162187  Streptomyces_cattleya_NRRL_8057___DSM_46488_uid77117  Streptomyces_coelicolor_A3_2__uid57801  Streptomyces_collinus_Tu_365_uid214429  Streptomyces_davawensis_JCM_4913_uid193657  Streptomyces_flavogriseus_ATCC_33331_uid40839  Streptomyces_fulvissimus_DSM_40593_uid201038  Streptomyces_griseus_NBRC_13350_uid58983  Streptomyces_hygroscopicus_jinggangensis_5008_uid89409  Streptomyces_hygroscopicus_jinggangensis_TL01_uid189753  Streptomyces_rapamycinicus_NRRL_5491_uid227224  Streptomyces_scabiei_87_22_uid46531  Streptomyces_venezuelae_ATCC_10712_uid177080  Streptomyces_violaceusniger_Tu_4113_uid52609  Streptosporangium_roseum_DSM_43021_uid42521  Sulfobacillus_acidophilus_DSM_10332_uid88061  Sulfobacillus_acidophilus_TPY_uid68841  Sulfolobus_acidocaldarius_DSM_639_uid58379  Sulfolobus_acidocaldarius_N8_uid189027  Sulfolobus_acidocaldarius_Ron12_I_uid189028  Sulfolobus_acidocaldarius_SUSAZ_uid232254  Sulfolobus_islandicus_HVE10_4_uid162067  Sulfolobus_islandicus_LAL14_1_uid197216  Sulfolobus_islandicus_L_D_8_5_uid43679  Sulfolobus_islandicus_L_S_2_15_uid58871  Sulfolobus_islandicus_M_14_25_uid58849  Sulfolobus_islandicus_M_16_27_uid58851  Sulfolobus_islandicus_M_16_4_uid58841  Sulfolobus_islandicus_REY15A_uid162071  Sulfolobus_islandicus_Y_G_57_14_uid58923  Sulfolobus_islandicus_Y_N_15_51_uid58825  Sulfolobus_solfataricus_98_2_uid167998  Sulfolobus_solfataricus_P2_uid57721  Sulfolobus_tokodaii_7_uid57807  Sulfuricella_denitrificans_skB26_uid170240  Sulfuricurvum_kujiense_DSM_16994_uid60789  Sulfurihydrogenibium_YO3AOP1_uid58855  Sulfurihydrogenibium_azorense_Az_Fu1_uid58121  Sulfurimonas_autotrophica_DSM_16294_uid53043  Sulfurimonas_denitrificans_DSM_1251_uid58185  Sulfurospirillum_barnesii_SES_3_uid168117  Sulfurospirillum_deleyianum_DSM_6946_uid41861  Sulfurovum_NBC37_1_uid58863  Symbiobacterium_thermophilum_IAM_14863_uid58165  Synechococcus_CC9311_uid58123  Synechococcus_CC9605_uid58319  Synechococcus_CC9902_uid58323  Synechococcus_JA_2_3B_a_2_13__uid58537  Synechococcus_JA_3_3Ab_uid58535  Synechococcus_PCC_6312_uid182934  Synechococcus_PCC_7002_uid59137  Synechococcus_PCC_7502_uid183008  Synechococcus_RCC307_uid61609  Synechococcus_WH_7803_uid61607  Synechococcus_WH_8102_uid61581  Synechococcus_elongatus_PCC_6301_uid58235  Synechococcus_elongatus_PCC_7942_uid58045  Synechocystis_PCC_6803_substr__GT_I_uid157913  Synechocystis_PCC_6803_substr__GT_I_uid158059  Synechocystis_PCC_6803_substr__PCC_N_uid159835  Synechocystis_PCC_6803_uid159873  Synechocystis_PCC_6803_uid189748  Synechocystis_PCC_6803_uid57659  Synergistetes_bacterium_SGP1_uid197182  Syntrophobacter_fumaroxidans_MPOB_uid58177  Syntrophobotulus_glycolicus_DSM_8271_uid63343  Syntrophomonas_wolfei_Goettingen_uid58179  Syntrophothermus_lipocalidus_DSM_12680_uid49527  Syntrophus_aciditrophicus_SB_uid58539  Tannerella_forsythia_ATCC_43037_uid83157  Taylorella_asinigenitalis_14_45_uid197194  Taylorella_asinigenitalis_MCE3_uid73771  Taylorella_equigenitalis_14_56_uid197193  Taylorella_equigenitalis_ATCC_35865_uid170255  Taylorella_equigenitalis_MCE9_uid62103  Tepidanaerobacter_Re1_uid66873  Tepidanaerobacter_acetatoxydans_Re1_uid184827  Teredinibacter_turnerae_T7901_uid59267  Terriglobus_roseus_DSM_18391_uid168183  Terriglobus_saanensis_SP1PR4_uid53251  Tetragenococcus_halophilus_uid74441  Thalassobaculum_L2_uid182483  Thalassolituus_oleivorans_MIL_1_uid195604  Thauera_MZ1T_uid58987  Thermacetogenium_phaeum_DSM_12270_uid177811  Thermaerobacter_marianensis_DSM_12885_uid61727  Thermanaerovibrio_acidaminovorans_DSM_6589_uid41925  Thermincola_potens_JR_uid48823  Thermoanaerobacter_X513_uid53065  Thermoanaerobacter_X514_uid58589  Thermoanaerobacter_brockii_finnii_Ako_1_uid55639  Thermoanaerobacter_italicus_Ab9_uid46241  Thermoanaerobacter_mathranii_A3_uid49481  Thermoanaerobacter_pseudethanolicus_ATCC_33223_uid58339  Thermoanaerobacter_tengcongensis_MB4_uid57813  Thermoanaerobacter_wiegelii_Rt8_B1_uid52581  Thermoanaerobacterium_thermosaccharolyticum_DSM_571_uid51639  Thermoanaerobacterium_thermosaccharolyticum_M0795_uid184821  Thermoanaerobacterium_xylanolyticum_LX_11_uid63163  Thermobacillus_composti_KWC4_uid74021  Thermobaculum_terrenum_ATCC_BAA_798_uid42011  Thermobifida_fusca_YX_uid57703  Thermobispora_bispora_DSM_43833_uid48999  Thermococcus_4557_uid70841  Thermococcus_AM4_uid54735  Thermococcus_CL1_uid168259  Thermococcus_barophilus_MP_uid54733  Thermococcus_gammatolerans_EJ3_uid59389  Thermococcus_kodakarensis_KOD1_uid58225  Thermococcus_litoralis_DSM_5473_uid82997  Thermococcus_onnurineus_NA1_uid59043  Thermococcus_sibiricus_MM_739_uid59399  Thermocrinis_albus_DSM_14484_uid46231  Thermodesulfatator_indicus_DSM_15286_uid68285  Thermodesulfobacterium_OPB45_uid68283  Thermodesulfobium_narugense_DSM_14796_uid66601  Thermodesulfovibrio_yellowstonii_DSM_11347_uid59257  Thermofilum_1910b_uid215374  Thermofilum_pendens_Hrk_5_uid58563  Thermogladius_1633_uid167488  Thermomicrobium_roseum_DSM_5159_uid59341  Thermomonospora_curvata_DSM_43183_uid41885  Thermoplasma_acidophilum_DSM_1728_uid61573  Thermoplasma_volcanium_GSS1_uid57751  Thermoplasmatales_archaeon_BRNA1_uid195930  Thermoproteus_tenax_Kra_1_uid74443  Thermoproteus_uzoniensis_768_20_uid65089  Thermosediminibacter_oceani_DSM_16646_uid51421  Thermosipho_africanus_TCF52B_uid59095  Thermosipho_melanesiensis_BI429_uid58683  Thermosphaera_aggregans_DSM_11486_uid48993  Thermosynechococcus_NK55_uid231517  Thermosynechococcus_elongatus_BP_1_uid57907  Thermotoga_RQ2_uid58935  Thermotoga_lettingae_TMO_uid58419  Thermotoga_maritima_MSB8_uid179902  Thermotoga_maritima_MSB8_uid202924  Thermotoga_maritima_MSB8_uid57723  Thermotoga_naphthophila_RKU_10_uid42777  Thermotoga_neapolitana_DSM_4359_uid59065  Thermotoga_petrophila_RKU_1_uid58655  Thermotoga_thermarum_DSM_5069_uid68449  Thermovibrio_ammonificans_HB_1_uid62095  Thermovirga_lienii_DSM_17291_uid77129  Thermus_CCB_US3_UF1_uid81197  Thermus_oshimai_JL_2_uid178948  Thermus_scotoductus_SA_01_uid62273  Thermus_thermophilus_HB27_uid58033  Thermus_thermophilus_HB8_uid58223  Thermus_thermophilus_JL_18_uid162129  Thermus_thermophilus_SG0_5JP17_16_uid159537  Thioalkalimicrobium_cyclicum_ALM1_uid67391  Thioalkalivibrio_K90mix_uid46181  Thioalkalivibrio_nitratireducens_DSM_14787_uid184011  Thioalkalivibrio_sulfidophilus_HL_EbGr7_uid59179  Thiobacillus_denitrificans_ATCC_25259_uid58189  Thiocystis_violascens_DSM_198_uid74025  Thioflavicoccus_mobilis_8321_uid184343  Thiomicrospira_crunogena_XCL_2_uid58183  Thiomonas_3As_uid178369  Thiomonas_intermedia_K12_uid48825  Tistrella_mobilis_KA081020_065_uid167486  Tolumonas_auensis_DSM_9187_uid59395  Treponema_azotonutricium_ZAS_9_uid67365  Treponema_brennaborense_DSM_12168_uid66607  Treponema_denticola_ATCC_35405_uid57583  Treponema_pallidum_Chicago_uid159543  Treponema_pallidum_DAL_1_uid87065  Treponema_pallidum_Fribourg_Blanc_uid201428  Treponema_pallidum_Mexico_A_uid176920  Treponema_pallidum_Nichols_uid208669  Treponema_pallidum_Nichols_uid57585  Treponema_pallidum_SS14_uid58977  Treponema_pallidum_pertenue_CDC2_uid87051  Treponema_pallidum_pertenue_Gauthier_uid87067  Treponema_pallidum_pertenue_SamoaD_uid87069  Treponema_paraluiscuniculi_Cuniculi_A_uid68447  Treponema_pedis_T_A4_uid215715  Treponema_primitia_ZAS_2_uid67367  Treponema_succinifaciens_DSM_2489_uid65781  Trichodesmium_erythraeum_IMS101_uid57925  Tropheryma_whipplei_TW08_27_uid57961  Tropheryma_whipplei_Twist_uid57705  Truepera_radiovictrix_DSM_17093_uid49533  Tsukamurella_paurometabola_DSM_20162_uid48829  Turneriella_parva_DSM_21527_uid168321  Ureaplasma_parvum_serovar_3_ATCC_27815_uid58887  Ureaplasma_parvum_serovar_3_ATCC_700970_uid57711  Ureaplasma_urealyticum_serovar_10_ATCC_33699_uid59011  Variovorax_paradoxus_B4_uid218005  Variovorax_paradoxus_EPS_uid62107  Variovorax_paradoxus_S110_uid59437  Veillonella_parvula_DSM_2008_uid41927  Verminephrobacter_eiseniae_EF01_2_uid58675  Verrucosispora_maris_AB_18_032_uid66297  Vibrio_EJY3_uid83161  Vibrio_Ex25_uid41601  Vibrio_alginolyticus_NBRC_15630___ATCC_17749_uid199933  Vibrio_anguillarum_775_uid68057  Vibrio_cholerae_IEC224_uid89389  Vibrio_cholerae_LMA3984_4_uid159541  Vibrio_cholerae_M66_2_uid59355  Vibrio_cholerae_MJ_1236_uid59387  Vibrio_cholerae_O1_2010EL_1786_uid78933  Vibrio_cholerae_O1_biovar_El_Tor_N16961_uid57623  Vibrio_cholerae_O395_uid159869  Vibrio_cholerae_O395_uid58425  Vibrio_fischeri_ES114_uid58163  Vibrio_fischeri_MJ11_uid58907  Vibrio_furnissii_NCTC_11218_uid82347  Vibrio_harveyi_ATCC_BAA_1116_uid218471  Vibrio_harveyi_ATCC_BAA_1116_uid58957  Vibrio_nigripulchritudo_SnF1_uid222819  Vibrio_parahaemolyticus_BB22OP_uid184822  Vibrio_parahaemolyticus_O1_K33_CDC_K4557_uid212977  Vibrio_parahaemolyticus_RIMD_2210633_uid57969  Vibrio_splendidus_LGP32_uid59353  Vibrio_vulnificus_CMCP6_uid62909  Vibrio_vulnificus_MO6_24_O_uid62243  Vibrio_vulnificus_YJ016_uid58007  Vulcanisaeta_distributa_DSM_14429_uid52827  Vulcanisaeta_moutnovskia_768_28_uid63631  Waddlia_chondrophila_WSU_86_1044_uid49531  Weeksella_virosa_DSM_16922_uid63627  Weissella_koreensis_KACC_15510_uid68837  Wigglesworthia_glossinidia_endosymbiont_of_Glossina_brevipalpis_uid57853  Wigglesworthia_glossinidia_endosymbiont_of_Glossina_morsitans__Yale_colony__uid88075  Wolbachia_endosymbiont_TRS_of_Brugia_malayi_uid58107  Wolbachia_endosymbiont_of_Culex_quinquefasciatus_Pel_uid61645  Wolbachia_endosymbiont_of_Drosophila_melanogaster_uid57851  Wolbachia_endosymbiont_of_Drosophila_simulans_wHa_uid198768  Wolbachia_endosymbiont_of_Drosophila_simulans_wNo_uid198767  Wolbachia_endosymbiont_of_Onchocerca_ochengi_uid171829  Wolbachia_wRi_uid59371  Wolinella_succinogenes_DSM_1740_uid61591  Xanthobacter_autotrophicus_Py2_uid58453  Xanthomonas_albilineans_GPE_PC73_uid43163  Xanthomonas_axonopodis_Xac29_1_uid193774  Xanthomonas_axonopodis_citri_306_uid57889  Xanthomonas_axonopodis_citrumelo_F1_uid73179  Xanthomonas_campestris_8004_uid57595  Xanthomonas_campestris_ATCC_33913_uid57887  Xanthomonas_campestris_raphani_756C_uid159539  Xanthomonas_campestris_uid61643  Xanthomonas_campestris_vesicatoria_85_10_uid58321  Xanthomonas_citri_Aw12879_uid194444  Xanthomonas_fuscans_4834_R_uid222814  Xanthomonas_oryzae_KACC_10331_uid58155  Xanthomonas_oryzae_MAFF_311018_uid58547  Xanthomonas_oryzae_PXO99A_uid59131  Xanthomonas_oryzae_oryzicola_BLS256_uid54411  Xenorhabdus_bovienii_SS_2004_uid46345  Xenorhabdus_nematophila_ATCC_19061_uid49133  Xylanimonas_cellulosilytica_DSM_15894_uid41935  Xylella_fastidiosa_9a5c_uid57849  Xylella_fastidiosa_GB514_uid162023  Xylella_fastidiosa_M12_uid58763  Xylella_fastidiosa_M23_uid58809  Xylella_fastidiosa_Temecula1_uid57869  Yersinia_enterocolitica_8081_uid57741  Yersinia_enterocolitica_palearctica_105_5R_r__uid63663  Yersinia_enterocolitica_palearctica_Y11_uid162069  Yersinia_pestis_A1122_uid158119  Yersinia_pestis_Angola_uid58485  Yersinia_pestis_Antiqua_uid58607  Yersinia_pestis_CO92_uid57621  Yersinia_pestis_D106004_uid158071  Yersinia_pestis_D182038_uid158073  Yersinia_pestis_KIM_10_uid57875  Yersinia_pestis_Nepal516_uid58609  Yersinia_pestis_Pestoides_F_uid58619  Yersinia_pestis_Z176003_uid47317  Yersinia_pestis_biovar_Medievalis_Harbin_35_uid158537  Yersinia_pestis_biovar_Microtus_91001_uid58037  Yersinia_pseudotuberculosis_IP_31758_uid58487  Yersinia_pseudotuberculosis_IP_32953_uid58157  Yersinia_pseudotuberculosis_PB1__uid59153  Yersinia_pseudotuberculosis_YPIII_uid59151  Zobellia_galactanivorans_uid70621  Zunongwangia_profunda_SM_A87_uid48073  Zymomonas_mobilis_ATCC_10988_uid55403  Zymomonas_mobilis_ATCC_29191_uid170612  Zymomonas_mobilis_CP4___NRRL_B_14023_uid229874  Zymomonas_mobilis_NCIMB_11163_uid41019  Zymomonas_mobilis_ZM4_uid58095  Zymomonas_mobilis_pomaceae_ATCC_29192_uid68445  _Cellvibrio__gilvus_ATCC_13127_uid68143  _Clostridium__sticklandii_uid59585  _Nostoc_azollae__0708_uid49725  _Ruminococcus__obeum_uid197165  _Ruminococcus__torques_uid197166  alpha_proteobacterium_HIMB59_uid175778  alpha_proteobacterium_HIMB5_uid175779  archaeon_Mx1201_uid196597  bacterium_BT_1_uid184079  beta_proteobacterium_CB_uid191340  butyrate_producing_bacterium_SM4_1_uid197180  butyrate_producing_bacterium_SS3_4_uid197159  butyrate_producing_bacterium_SSC_2_uid197181  candidate_division_SR1_bacterium_RAAC1_SR1_1_uid230714  candidate_division_WWE3_bacterium_RAAC2_WWE3_1_uid230713  cyanobacterium_UCYN_A_uid43697  delta_proteobacterium_BABL1_uid230716  gamma_proteobacterium_HdN1_uid51635  halophilic_archaeon_DL31_uid72619  secondary_endosymbiont_of_Ctenarytaina_eucalypti_uid172737  secondary_endosymbiont_of_Heteropsylla_cubana_Thao2000_uid172738  syncytium_symbiont_of_Diaphorina_citri_uid213384  uncultured_Sulfuricurvum_RIFRC_1_uid193658  uncultured_Termite_group_1_bacterium_phylotype_Rs_D17_uid59059 |
| --- |
